# Naringenin chalcone carbon double-bond reductases mediate dihydrochalcone biosynthesis in apple leaves

**DOI:** 10.1093/plphys/kiae515

**Published:** 2024-09-29

**Authors:** Yar-Khing Yauk, Andrew P Dare, Janine M Cooney, Yule Wang, Cyril Hamiaux, Tony K McGhie, Mindy Y Wang, Pengmin Li, Ross G Atkinson

**Affiliations:** The New Zealand Institute for Plant and Food Research Limited (Plant and Food Research), Auckland 1142, New Zealand; The New Zealand Institute for Plant and Food Research Limited (Plant and Food Research), Auckland 1142, New Zealand; Plant and Food Research, Hamilton 3240, New Zealand; State Key Laboratory for Crop Stress Resistance and High-Efficiency Production/Shaanxi Key Laboratory of Apple, College of Horticulture, Northwest A&F University, Yangling, Shaanxi 712100, China; The New Zealand Institute for Plant and Food Research Limited (Plant and Food Research), Auckland 1142, New Zealand; Plant and Food Research, Palmerston North 4442, New Zealand; The New Zealand Institute for Plant and Food Research Limited (Plant and Food Research), Auckland 1142, New Zealand; State Key Laboratory for Crop Stress Resistance and High-Efficiency Production/Shaanxi Key Laboratory of Apple, College of Horticulture, Northwest A&F University, Yangling, Shaanxi 712100, China; The New Zealand Institute for Plant and Food Research Limited (Plant and Food Research), Auckland 1142, New Zealand

## Abstract

Dihydrochalcones (DHCs) are flavonoids produced as a side branch of the phenylpropanoid pathway. DHCs are found at high concentrations in apples (*Malus* spp.) but not in pears (*Pyrus* spp.) or other members of the Rosaceae. Biosynthesis of DHCs in apple has been hypothesized to occur via reduction of *p*-coumaroyl CoA by a *Malus* × *domestica* hydroxycinnamoyl CoA double-bond reductase (MdHCDBR) followed by the action chalcone synthase to produce phloretin or via direct reduction of naringenin chalcone to phloretin via an unknown enzyme. In this study, we report that genetic downregulation of *MdHCDBR* does not reduce DHC concentrations in apple leaves. We used comparative transcriptome analysis to identify candidate naringenin chalcone reductases (NCRs), designated *MdNCR1a*–*c*, expressed in apple leaves but not fruit. These *MdNCR1* genes form an expanded gene cluster found exclusively in apple. Transient expression of *MdNCR1* genes in *Nicotiana benthamiana* leaves indicated they produced DHCs at high concentrations in planta. Recombinant MdNCR1 utilized naringenin chalcone to produce phloretin at high efficiency. Downregulation of NCR genes in transgenic apple reduced foliar DHC levels by 85% to 95%. Reducing DHC production redirected flux to the production of flavonol glycosides. In situ localization indicated that NCR proteins were likely found in the vacuolar membrane. Active site analysis of AlphaFold models indicated that MdNCR1a–c share identical substrate binding pockets, but the pockets differ substantially in related weakly active/inactive NCR proteins. Identifying the missing enzyme required for DHC production provides opportunities to manipulate DHC content in apple and other fruits and has other applications, e.g. in biofermentation and biopharming.

## Teaser

Carbon double-bond reductases reduce naringenin chalcone to produce phloretin in the dihydrochalcone biosynthesis pathway in apple leaves.

## Introduction

**Figure 1.**
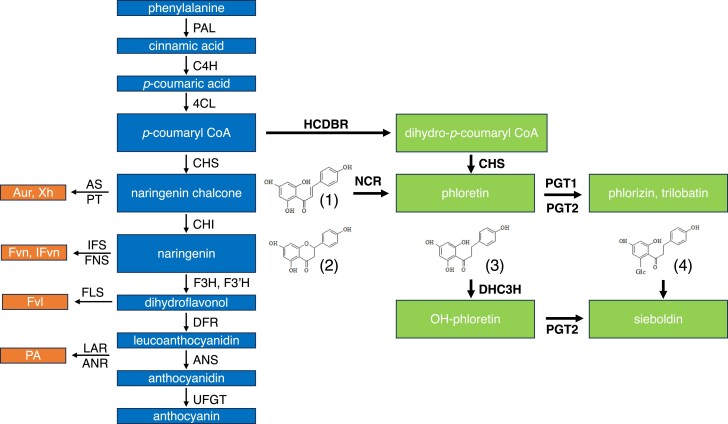
Pathways to DHC biosynthesis in apple leaves. The flavonoid pathway is shown in blue. PAL, phenylalanine ammonia lyase; C4H, cinnamate 4-hydroxylase; 4CL, 4-coumaroyl ligase; CHS, chalcone synthase; CHI, chalcone isomerase; F3H, flavanone 3-hydroxylase; F3ʹH, flavonoid 3′-hydroxylase; DFR, dihydroflavonol reductase; ANS, anthocyanidin synthase; UFGT, UDP-glucose flavonoid 3-*O*-glucosyltransferase. Selected side branches toward production of aurones (Aur), xanthohumol (Xh), flavones (Fvn), isoflavones (IFvn), flavonols (Fvl), and proanthocyanidins (PA) are shown in orange. AS, aureusidin synthase; PT, prenyltransferase; IFS, isoflavone synthase; FNS, flavanone synthase; FLS, flavonol synthase; LAR, leucoanthocyanidin reductase; ANR, anthocyanidin reductase. The pathways to production of DHCs are shown in green and the enzymes involved are in bold. HCDBR, hydroxycinnamoyl-CoA carbon double-bond reductase; NCR, naringenin chalcone reductase; PGT1, phloretin glycosyltransferase1; PGT2, phloretin glycosyltransferase2; DHC3H, dihydrochalcone 3-hydroxylase. Compound structures: (1) naringenin chalcone; (2) naringenin; (3) phloretin; (4) phlorizin.

The adaptation of plants to a terrestrial environment 400 million years ago brought with it the need to develop specialized metabolites to cope with the challenges of living on dry land. The emergence of the flavonoid pathway allowed the production of a range of phenolic compounds derived from a phenylalanine precursor. These compounds included structural polymers like lignin and UV-absorbing flavonoids, which enabled angiosperms to colonize multiple environments on land (de Vries et al. 2021). Plants contain more than 4,000 compounds derived from the flavonoid pathway, with this diversity being in part due to the number of side branches, which use intermediates from a conserved “core pathway” (Fig. 1). Dihydrochalcones (DHCs) are a class of flavonoids that consist of phenolic rings linked by a saturated three carbon bridge. An unusual structural feature of DHCs is their open C ring configuration and the absence of a double bond in the α-β position, which plays a critical role in the cyclization of naringenin chalcone to form the classical A, B and C ring structure of flavonoids. The best known DHC is phlorizin (phloretin-2ʹ-*O*-glucoside), which is present in almost all apple (*Malus* × *domestica*) tissues, but is highest in the leaves where it forms up to 18% of the leaf dry weight (Mikulic Petkovsek et al. 2009). Dietary phlorizin intake ranges from 0.7 to 7.5 mg daily (Niederberger et al. 2020) and is linked to a number of human health benefits (Ehrenkranz et al. 2005), largely bestowed by the consumption of apples and apple products. The DHC trilobatin (phloretin-4ʹ-*O*-glucoside) is a natural low calorie sweetener that is found at high concentrations in the leaves of a range of crab apples (*Malus* spp.) (Wang et al. 2020). Pears (*Pyrus* spp.), although closely related to apple (Wu et al. 2013), do not produce detectable quantities of DHCs.

Despite their importance to human health and discovery over 100 years ago, the pathway to DHC production in apple remains only partially understood. Early studies on phlorizin biosynthesis using radiolabeled hydroxycinnamic acids suggested a branch point below *p*-coumaric acid (Avadhani and Towers 1961). A much later study using apple leaf extracts incubated with ^13^C labeled substrates suggested that the DHC side branch occurred at *p*-coumaroyl CoA via the action of a carbon double-bond reductase (CDBR) to form dihydro-*p*-coumaroyl CoA, which was subsequently converted to phloretin in vitro by the action of chalcone synthase (CHS) (Gosch et al. 2009). Phloretin is converted to phlorizin or trilobatin by the action of the UDP-glycosyltransferases *MdPGT1* (Jugdé et al. 2008) and *MdPGT2* (Wang et al. 2020), respectively. To date, two classes of CDBRs from *Malus* have been described in the literature. Two enoyl CoA reductase-like gene homologs (MdENRL-3/MdENRL-5) were identified by Dare et al. (2013), which produced phlorizin when transiently expressed in tobacco (*Nicotiana tabacum*). However, RNAi silencing of these genes failed to produce significant reductions in foliar DHC concentrations in multiple transgenic lines (Dare et al. 2013). A hydroxycinnamoyl CoA double-bond reductase (MdHCDBR), with high similarity to the raspberry ketone zingerone synthase (RZS-1) involved in raspberry ketone formation, was subsequently shown to reduce the α-β double bond in *p*-coumaroyl CoA in in vitro reactions (Ibdah et al. 2014), however, in planta evidence to support its role in *Malus* is yet to emerge.

A second potential branchpoint to produce DHCs in apple is a one-step reduction of naringenin chalcone to phloretin by a naringenin chalcone double-bond reductase (NCR) (Fig. 1). To date, only a few pathways have been described as originating at this point: the formation of aurones by the action of a glycosyltransferase and an aureusidin synthase (Ono et al. 2006), auronidins derived from aurones (Berland et al. 2019), and the formation of xanthohumol using prenylation and *O*-methylation of the chalcone scaffold (Ban et al. 2018). The rapid cyclization of naringenin chalcone to naringenin by chalcone isomerase (CHI) means that to divert sufficient flux to phloretin synthesis, there must be low CHI activity and the NCR must have very high catalytic efficiency or be very highly expressed. A previous study reported comparatively low CHI activity in “Royal Gala” leaves, which was overcome with the overexpression of an *AtCHI* transgene and resulted in up to a 19-fold decrease in phlorizin levels, presumably as a result of the depletion of the naringenin chalcone substrate pool (Dare et al. 2020). The presence of an NADPH-dependent NCR activity was also demonstrated in “Royal Gala” leaf extracts, inferring naringenin chalcone may be a potential entry point for the phlorizin pathway in apple leaves, however, the exact nature of the enzyme activity remained undescribed (Dare et al. 2020).

In response to pathogen attack or as a result of oxidative stress within cellular organelles such as the chloroplast, plants generate high reactive oxidized species or “oxylipins” derived from peroxidated polyunsaturated fatty acids (Curien et al. 2016). Of the oxylipins, those containing unsaturated α-β carbonyl groups are considered the most reactive because of their ability to form adducts with amino acids and nucleic acids. The quinone oxidoreductase (QR) family belongs to the medium chain dehydrogenase reductase superfamily and is one class of enzyme capable of detoxifying reactive oxylipins by reducing the carbon double bond in the α-β carbonyl group. The chloroplast envelope QR from Arabidopsis (At4g13010, AtceQORH) is encoded by the nuclear genome and transported via an alternative import pathway to the inner chloroplast membrane (Curien et al. 2016). A previous investigation into the substrate range of AtceQORH showed preference for long chain α-β unsaturated ketones, particularly gamma ketols that are produced by jasmonic acid biosynthesis. Intriguingly, they also showed a high catalytic efficiency in reducing the carbon double bond in synthetic unsaturated ketone trans 1,3 diphenyl propen-2-ene-1-one (*trans*-chalcone). This has the same basic chalcone scaffold as naringenin chalcone (2ʹ,4,4ʹ,6ʹ-tetrahydroxychalcone) and a carbon bond located in an analogous position, adjacent to the carbonyl group.

In this study, we identified three apple QR-like genes (*MdNCR1a*–*c*) capable of producing DHCs when transiently expressed in *Nicotiana benthamiana*. Recombinant MdNCR1 performed NADPH-dependent reduction of naringenin chalcone to phloretin in vitro, and RNAi-mediated silencing of the NCR genes in transgenic apple resulted in up to 95% reduction in foliar DHC levels. Our results indicate the side branch leading to DHC biosynthesis in *Malus* occurs primarily at naringenin chalcone via direct reduction to phloretin and not at *p*-coumaroyl CoA via reduction to dihydro-*p*-coumaroyl CoA.

## Results

### Downregulation of *MdHCDBR* in apple leaves

The NADPH-dependent *MdHCDBR* has previously been implicated in the production of DHCs in vitro, because recombinant MdHCDBR can reduce *p*-coumaroyl CoA to dihydro-*p*-coumaroyl CoA (Ibdah et al. 2014). Evidence for the ability of *MdHCDBR* to produce DHCs in *N. benthamiana* leaves when co-expressed with *MdCHS* and *MdPGT1/MdPGT2* was subsequently shown by Wang et al. (2020). To establish the importance of *MdHCDBR* (MD15G1145700) in DHC production in apple leaves, we generated an RNA-interference (RNAi) construct to reduce the expression of *MdHCDBR* and a second putative CDBR, *MdARL2* (Alkenal Reductase Like; MD12G1260900), present in the *Malus* genome (Supplementary Fig. S1), which was expressed in apple leaves too (Supplementary Table S1A). Two additional homologs of *MdHCDBR* (*MdARL1*—MD02G1002400 and *MdARL3*—MD02G1001800), also expressed in leaves (Supplementary Table S1A) might also be expected to be downregulated by the RNAi construct. These homologs show >93% nucleotide identity to *MdHCDBR* (Supplementary Table S1B).

Twelve independent “Royal Gala” transgenic lines were generated by *Agrobacterium*-mediated transformation and Reverse transcription-quantitative polymerase chain reaction (RT-qPCR) analysis of these lines indicated that expression of both *MdHCDBR* and *MdARL2* were downregulated by >90% in six lines (Supplementary Fig. S2A). HPLC analysis of the six knockdown transgenic lines showed no reduction in the concentrations of foliar phlorizin (Supplementary Fig. S2B). Expression of *MdARL1* and *MdARL3* was assessed in four of the knockdown lines and was also significantly reduced (Supplementary Fig. S2C). From this experiment, we concluded that *MdHCDBR* and *MdARL1* to 3 are not responsible for the high concentrations of DHCs in apple leaves.

### Identification of candidate NCRs in apple

An alternative pathway to DHC production in apple has been suggested to occur via direct reduction of naringenin chalcone to phloretin via a NCR (Dare et al. 2020). To identify candidate NCR genes, a comparative transcriptomics approach was undertaken between apple leaves and fruit. Gene models with descriptors containing words such as reductase and dehydrogenase were screened to identify those showing higher expression in leaves compared with fruit (Supplementary Table S2). The rationale was that expression of different CDBR genes might account for the >100× higher level of DHCs produced in apple leaves (mg·g^−1^) compared with fruit (µg·g^−1^). Secondary screening conditions were that expression was also found in stems and very young fruit, matching the tissues where NCR activity in apple had been reported (Dare et al. 2020). Based on these criteria, three gene models (MD05G1167400, MD05G1167600, and MD05G1168200) annotated as “putative chloroplastic quinone-oxidoreductase homologs” were identified as lead candidate NCRs.

**Figure 2.**
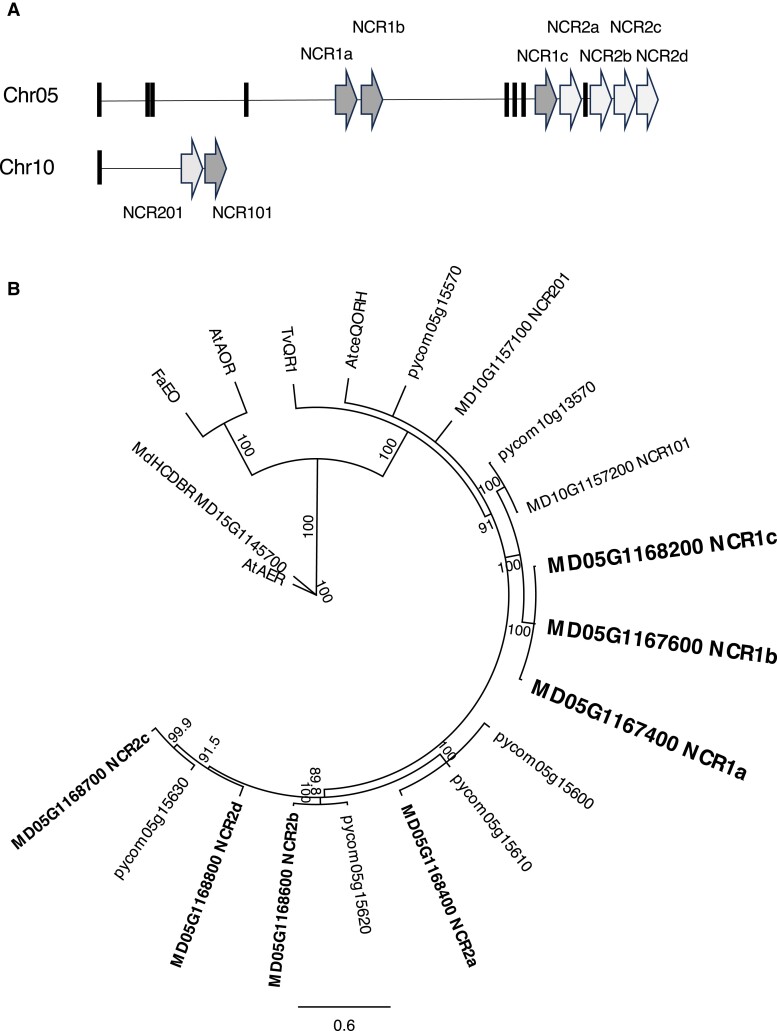
NCR genomic organization and phylogenetic analysis. **A)** Genomic organization of NCR gene models on Chr 05 and 10 from the “Golden Delicious” apple genome assembly (Daccord et al. 2017). NCR gene models with full-length open Reading frames are shown as arrows and likely pseudogenes as vertical black bars. The genomic location, open Reading frame size and annotation of each gene model are listed in Supplementary Table S3. **B)** Phylogram of NCR gene models from apple and pear and previously characterized quinone reductases. Amino acid alignments were generated with the MUSCLE alignment tool in Geneious Prime (Version 2022.0.1). Trees were inferred using the Maximum Likelihood method based on the JTT matrix-based model (Jones et al. 1992). Percentage bootstrap values >80% (1,000 replicates) are shown. Branch lengths measure the number of substitutions per site, relative to the scale bar. Genes in the *MdNCR1* clade specific to apple are shown in larger font size and bolded. Genes in the paralogous *MdNCR2* clade on Chr 05 are bolded. Apple (MD) and pear (pycom) gene models were obtained from Genome Database for Rosaceae (Jung et al. 2019). *AtceQORH* (Q9SV68.1), *TvQR1* (Q9AYU1.1) *AtAOR* (Q9ZUC1.2), *FaEO* (Q941I0.2), *MdHCDBR* (MD15G1145700), and *AtAER* (Q39172.1).

These three gene models formed part of a cluster of 15 related gene models on chromosome (Chr) 05 (Fig. 2A). Seven of the gene models on Chr 05 encoded likely full-length open reading frames (ORFs; 329 to 334 amino acids, aa), while eight encoded pseudogenes with shortened ORFs (54 to 228 aa; Supplementary Table S3). Three related gene models also annotated as chloroplastic quinone-oxidoreductase homologs were located on homoeologous Chr 10 (Fig. 2A), with two of these gene models encoding full-length ORFs of 329 aa (Supplementary Table S3). MD05G1167400, MD05G1167600, and MD05G1168200 were highly related to each other (>98% amino acid identity, Supplementary Fig. S3A) and were designated *MdNCR1a*, *1b,* and *1c*, respectively (Supplementary Table S3). The three *MdNCR1* genes showed ∼86% amino acid identity to gene model MD10G1157200 on Chr 10 designated *MdNCR101*. The remaining NCR gene models on Chr 05 and Chr 10 were designated *MdNCR2a–d* and *MdNCR201*, respectively, and showed lower levels of amino acid identity (72% to 77%) to *MdNCR1a–c* (Supplementary Table S4A).

Phylogenetic analysis (Fig. 2B) indicated that the apple NCR genes are most closely related to *AtceQORH* (*A. thaliana* chloroplast envelope QR homolog) and *TvQR1* (*Triphysaria versicolor* QR 1), and more distantly related to other reductases, such as *AtAOR* (*A. thaliana* alkenal oxidoreductase), *FaEO* (*Fragaria* × *ananassa* oxidoreductase) and *AtAER/P1-ZCr* (*A. thaliana* alkenal oxidoreductase/z-crystallin protein). Comparison of apple NCR genes to gene models in the *Pyrus communis* genome indicated that the *MdNCR1a–c* gene models formed a clade unique to apple, without an orthologous pear gene model equivalent (Fig. 2B). Further phylogenetic analysis indicated that all seven sequenced *Malus* spp. genomes (listed in Supplementary Fig. S3B) contain members of the NCR1 clade. The nearest NCR1 gene models in five sequenced *Pyrus* spp. genomes (listed in Supplementary Fig. S3B) and other close relatives in the Maleae, such as Chinese hawthorn (*Craetagus pinnatifida*) and loquat (*Eriobotrya japonica*), are NCR101-like. *Gillenia trifoliata*, a member of the Gillenieae clade (x = 9) that is the basal clade to the Maleae (x = 17) before a whole genome duplication event (Xiang et al. 2017), also contains an NCR101-like gene model (Supplementary Fig. S3B).

### Transient expression of candidate NCRs

**Figure 3.**
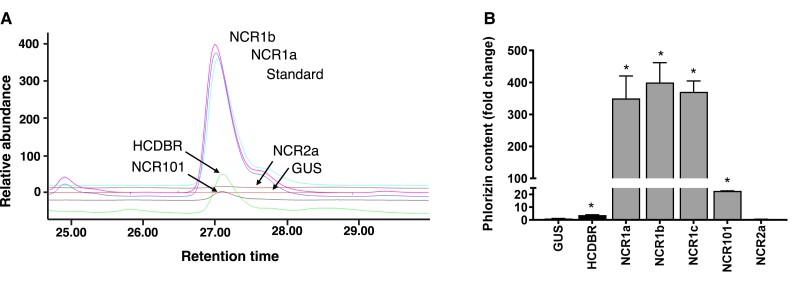
Transient expression of apple NCR genes in planta. **A)** Representative HPLC traces of inoculations into *N. benthamiana*. All inoculations were performed in combination with pHEX2_PGT1, pHEX2_CHS, and pHEX2_MYB10. Leaves were inoculated with pHEX2_GUS as a negative control and pHEX2_HCDBR as a positive control. *MdNCR1b* (purple, 5× dilution), *MdNCR1a* (dark blue, 5× dilution), phlorizin standard (light blue), *MdNCR2a* (brown)*, GUS* (red), *MdNCR101* (black, 5× dilution), and *MdHCDBR* (green). **B)** Relative production of phlorizin in *N. benthamiana* leaves inoculated with *MdHCDBR* (black bar) and the five NCR genes (gray bars) compared to the *GUS* control (set at 1). Data are mean ± SE (*n* = 3 independent inoculations). Statistical analysis in GraphPad Prism: *t*-test vs GUS control, *P* < 0.05 = *.

Five unique NCR cDNAs were successfully cloned into the transient expression vector pHEX2: *MdNCR1a* corresponding to MD05G1167400, *MdNCR1b* to MD05G1167600, *MdNCR1c* to MD05G1168200, *MdNCR101* to MD10G1157200, and *MdNCR2a* to MD05G1168400. Each binary vector construct was coinfiltrated into *N. benthamiana* leaves with a pHEX2_PGT1 construct that converts the highly reactive DHC phloretin into stable glycosylated phlorizin (Fig. 1). Extracts from leaves infiltrated with the pHEX2_GUS control construct or with pHEX2_NCR2a produced negligible amounts of phlorizin (Fig. 3A). The positive control pHEX2_HCDBR produced low but detectable amounts of phlorizin (150 μg·g^−1^ fresh weight, ∼4-fold greater than the GUS control), confirming previously published results (Wang et al. 2020). Leaves inoculated with pHEX2_NCR101 produced >20× the phlorizin produced by the GUS control. pHEX2_NCR1a–c constructs produced significantly higher amounts of phlorizin (>300×, 15 to 20 mg·g^−1^ fresh weight, Fig. 3B) compared with GUS and HCDBR controls (Fig. 3, A and B, Supplementary Fig. S3C). This result indicated that *MdNCR1a*–*c* could each mediate the production of high concentrations of DHCs in planta.

### Characterization of recombinant MdNCR1b

**Figure 4.**
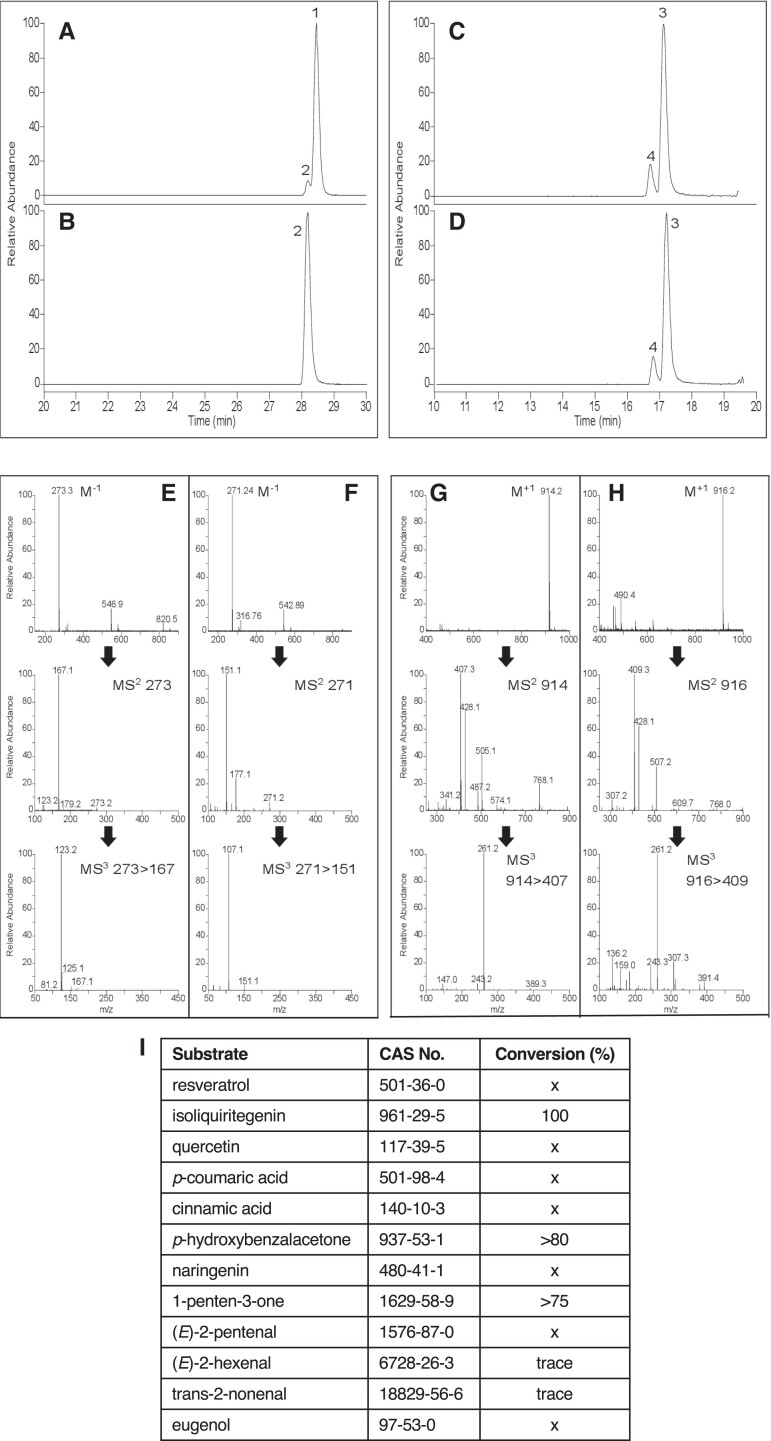
Activity of recombinant MdNCR1b in vitro. Recombinant MdNCR1b and MdHCDBR proteins were expressed in *E. coli*, purified by Ni^2+^ affinity and gel filtration chromatography, and reductase activity vs naringenin chalcone and *p*-coumaroyl-CoA assessed by LC-ESI-MS in negative mode. Base peak chromatogram: **A)** MdNCR1b + naringenin chalcone, **B)** MdHCDBR + naringenin chalcone, **C)** MdNCR1b + *p*-coumaroyl-CoA, **D)** MdHCDBR + *p*-coumaroyl-CoA; mass spectrometry (MS) spectra and fragmentation patterns: **E)** fullscan (M^−1^), MS^2^, and MS^3^ for peak 1 (phloretin), **F)** fullscan (M^−1^), MS^2^, and MS^3^ for peak 2 (naringenin), **G)** fullscan (M^−1^), MS^2^, and MS^3^ for peak 3 (*p*-coumaroyl-CoA), **H)** fullscan (M^−1^), MS^2^, and MS^3^ for peak 4 (dihydro *p*-coumaroyl-CoA). **I)** Activity of MdNCR1b toward additional substrates containing carbon–carbon double bonds. Assays were replicated twice. x = no activity detected.

As *MdNCR1a*–*c* show >98% amino acid identity and exhibit essentially identical activity when transiently expressed in planta, one protein, MdNCR1b, was selected for detailed biochemical analysis. MdNCR1b was expressed as His-tagged recombinant proteins in *Escherichia coli* and purified by gel filtration chromatography (Supplementary Fig. S4A). MdNCR1b readily reduced naringenin chalcone to form phloretin in the presence of NADPH, but very weakly when supplied with NADH. The conversion of naringenin chalcone to phloretin by MdNCR1b is shown in Fig. 4A (peak 1). A trace amount of naringenin (peak 2) was also observed, resulting from spontaneous isomerization of naringenin chalcone to naringenin, as previously reported (Mol et al. 1985). MdHCDBR was not able to reduce naringenin chalcone to phloretin and only naringenin was detected after incubation (Fig. 4B). MdNCR1b also catalyzed the reduction of ∼18% of supplied *p*-coumaroyl CoA (peak 3) to dihydro-*p*-coumaroyl CoA (peak 4) (Fig. 4C). This conversion occurred at a similar efficiency to that mediated by MdHCDBR (Fig. 4D). MS analysis confirmed the identity of each peak (Fig. 4, E to H). Phloretin was detected as a M^−1^ peak of *m/z* 273, showing diagnostic MS2 and MS3 fragmentation ions *m/z* 167 and *m/z* 123, respectively (Fig. 4E). Naringenin and naringenin chalcone showed identical collision induced fragmentation patterns, but could be separated and distinguished chromatographically, with the chalcone structural isomer eluting slightly later under reverse phase conditions. Naringenin gave an M^−1^ peak of *m/z* 271, yielding the expected major MS2 ion *m/z* 151 via retro Diels Alder cleavage of the C-ring, followed by subsequent loss of CO_2_ in the MS3 to give *m/z* 107 (Fig. 4F). For *p*-coumaroyl CoA and dihydro-*p*-coumaroyl CoA M ^+ 1^ ions of *m/z* 914 and 916 were observed, respectively. Both gave the predicted MS2 fragmentation pattern expected for CoA species (Jones et al. 2021), with cleavage of the CoA moiety at the 3-phosphate-adenosine-5ʹ-diphosphate giving rise to a unique product ion following neutral loss of *m/z* 507 (*m/z* 407 or 409) and a common daughter ion *m/z* 428 arising from further fragmentation of the phosphate-adenosine portion between the 5ʹ-diphosphates.

The activity of MdNCR1b was tested toward a range of other phenolic and volatile substrates by LC-MS and GC-MS (Fig. 4I). MdNCR1b showed the ability to reduce isoliquiritigenin to davidigenin and *p*-hydroxybenzalacetone to raspberry ketone. Of the five volatile substrates tested, MdNCR1b showed reductase activity toward 1-penten-3-one and trace activity toward (*E*)-2-hexenal and trans-2-nonenal.

MdNCR1b showed reductase activity toward naringenin chalcone over a broad temperature range (25 °C to 55 °C) with maximum activity between 30 °C and 40 °C (Supplementary Fig. S4B). Naringenin chalcone isomerization was stable between 25 °C to 40 °C and was slightly higher at 45 °C to 55 °C. MdNCR1b reductase activity decreased from pH 3.0 to 5.0 and increased slightly at pH 6.0. Isomerization of naringenin chalcone was highest at pH 6.0. (Supplementary Figure S4C). Kinetic parameters were determined for MdNCR1b with respect to naringenin chalcone and NADPH. Reactions were performed at 30 °C at pH 3.0 for 30 min to minimize isomerization of naringenin chalcone to naringenin. (Supplementary Figure S4C). MdNCR1b showed a *K*_m_ of 9.9 ± 2.6 µm for naringenin chalcone (Supplementary Fig. S4D) with a catalytic efficiency (*k*_cat_/*K*_m_) of 18.5 s^−1^·mM^−1^. The observed K_m_ for NADPH was 23.1 ± 9.4 µm (Supplementary Fig. S4E) with a catalytic efficiency of 3.1 s^−1^·mM^−1^.

### Expression analysis of apple NCR genes by RT-qPCR

**Figure 5.**
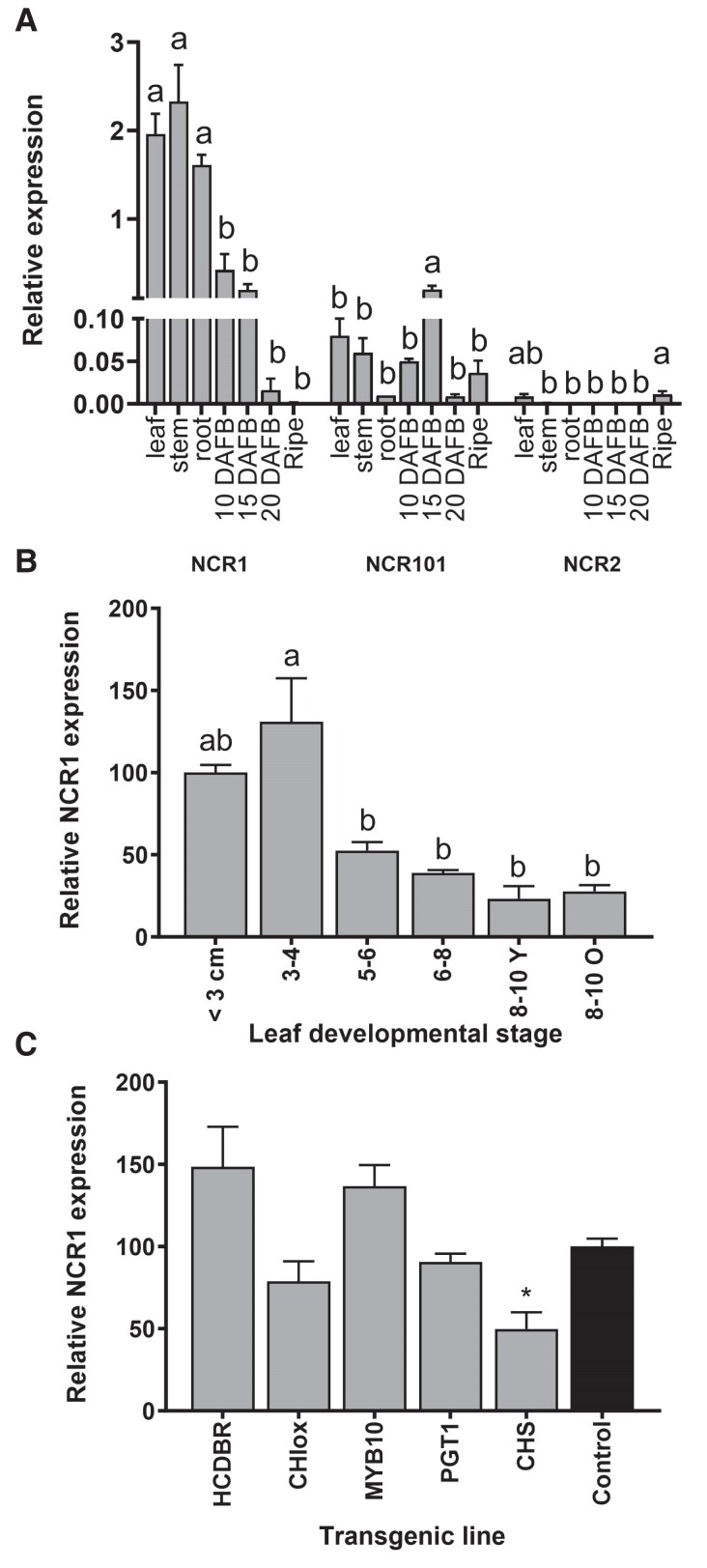
Relative expression of NCR genes in apple tissues. **A)** Relative gene expression of *MdNCR1*, *MdNCR101*, and *MdNCR2* were determined by qRT-PCR in different tissues leaf (5 to 6 cm), stem, root, young fruit 10, 15, and 20 DAFB and ripe fruit (165 DAFB). Primers are given in Supplementary Table S5. **B)***MdNCR1* expression during leaf development from <3 to 8 to 10 cm in length. 8 to 10 Y = fully expanded mature leaves. 8 to 10 O = fully expanded older leaves. For **A)** and **B)** Statistical analysis in GraphPad Prism: one-way ANOVA using Tukey's correction for multiple comparisons. Means with the same letter were not significantly different at the *P* < 0.05 level. **C)***MdNCR1* expression in transgenic apple leaves downregulated for *MdHCDBR* (Hn2.2, Supplementary Fig. S2), *MdPGT1* (line A17; Dare et al. 2017), *MdCHS* (line A9; Dare et al. 2013) or overexpressing Arabidopsis *CHI* (line A7; Dare et al. 2020) or *MdMYB10* (line A4; Espley et al. 2013). The control was WT “Royal Gala.” Data are presented as means ± SE (*n* = 3 biological replicates). Statistical analysis in GraphPad Prism: one-way ANOVA using Dunnett's Multiple Comparison Test vs control, *P* < 0.1 = *.

To explore the expression of NCR genes in apple, specific primers were designed that would amplify *MdNCR1* (*MdNCR1a*–*c* expression together), *MdNCR101* and *MdNCR2a*. *MdNCR1* expression was highest in leaves, stems, and roots, and ∼10-fold lower in young fruitlets at 10 and 15 days after full bloom (DAFB) (Fig. 5A). Barely detectable levels of expression were observed in fruitlets by 20 DAFB and no expression was measured in ripe fruit. Low level expression of *MdNCR101* was detected in all tissues and *MdNCR2* expression was essentially zero. These results support the criteria originally used to select candidate NCR1 genes using transcriptome data in the Apple Multidimensional Omics Database (Da et al. 2019).

Expression of *MdNCR1* during leaf development was highest early in leaf development (<4 cm) and decreased as leaves expanded. Expression was similar in young fully expanded leaves and older mature leaves (Fig. 5B). This expression pattern was similar to that previously reported for accumulation of phlorizin during leaf development (Picinelli et al. 1995) and in studies showing that phlorizin concentrations are generally stable in mature leaves during the growing season (Mikulic Petkovsek et al. 2009).

*MdNCR1* expression was also tested in a range of key transgenic plants in which other flavonoid pathway genes had been manipulated (Fig. 5C). *MdNCR1* expression was unaffected in the transgenic lines downregulated for *MdHCDBR/MdARL2* described in Supplementary Fig. S2. *MdNCR1* was also unchanged in the *MdMYB10* overexpressing line A7, which showed a general increase in flux through the flavonoid pathway (Espley et al. 2013). *MdNCR1* expression did not change in transgenic plants where the pool of MdNCR1 product (phloretin) was expected to accumulate (*MdPGT1* downregulated line A17; Dare et al. 2017) and in plants where the pool of substrate (naringenin chalcone) was reduced by heterologous expression of an Arabidopsis *CHI* (CHIox line A7; Dare et al. 2020). A 50% reduction in *MdNCR1* expression was observed in the *MdCHS* downregulated line A9 (Dare et al. 2013) in which accumulation of DHCs and flavonoids was reduced by >90%. In these plants, the reduction in *MdNCR1* expression may be a response to reduction in available naringenin chalcone precursor or to the increase in compounds lower in flavonoid pathway.

### Downregulation of NCR genes in transgenic apple leaves

To establish the importance of NCR genes in DHC production in apple leaves, a concatenated RNAi construct was generated to downregulate *MdNCR1a–c* and *MdNCR101*. Nine independent transgenic lines were regenerated via *Agrobacterium*-mediated transformation and initially screened in tissue culture for *MdNCR1* gene expression by RT-qPCR (Supplementary Fig. S5A) and for phlorizin concentration in leaves by HPLC (Supplementary Fig. S5B). Four lines—NT5, 10, 14, and 21—were chosen for further detailed analysis, which were micro-grafted onto rootstocks and grown in a containment glasshouse alongside untransformed “Royal Gala” controls. Transgenic lines showed normal growth and development with no changes in leaf size or internode length. The NCR transgenics had normal morphology and architecture, in contrast to the transgenic lines downregulated for *MdCHS* (Dare et al. 2013) and *MdPGT1* (Dare et al. 2017), which showed severe developmental abnormalities.

**Figure 6.**
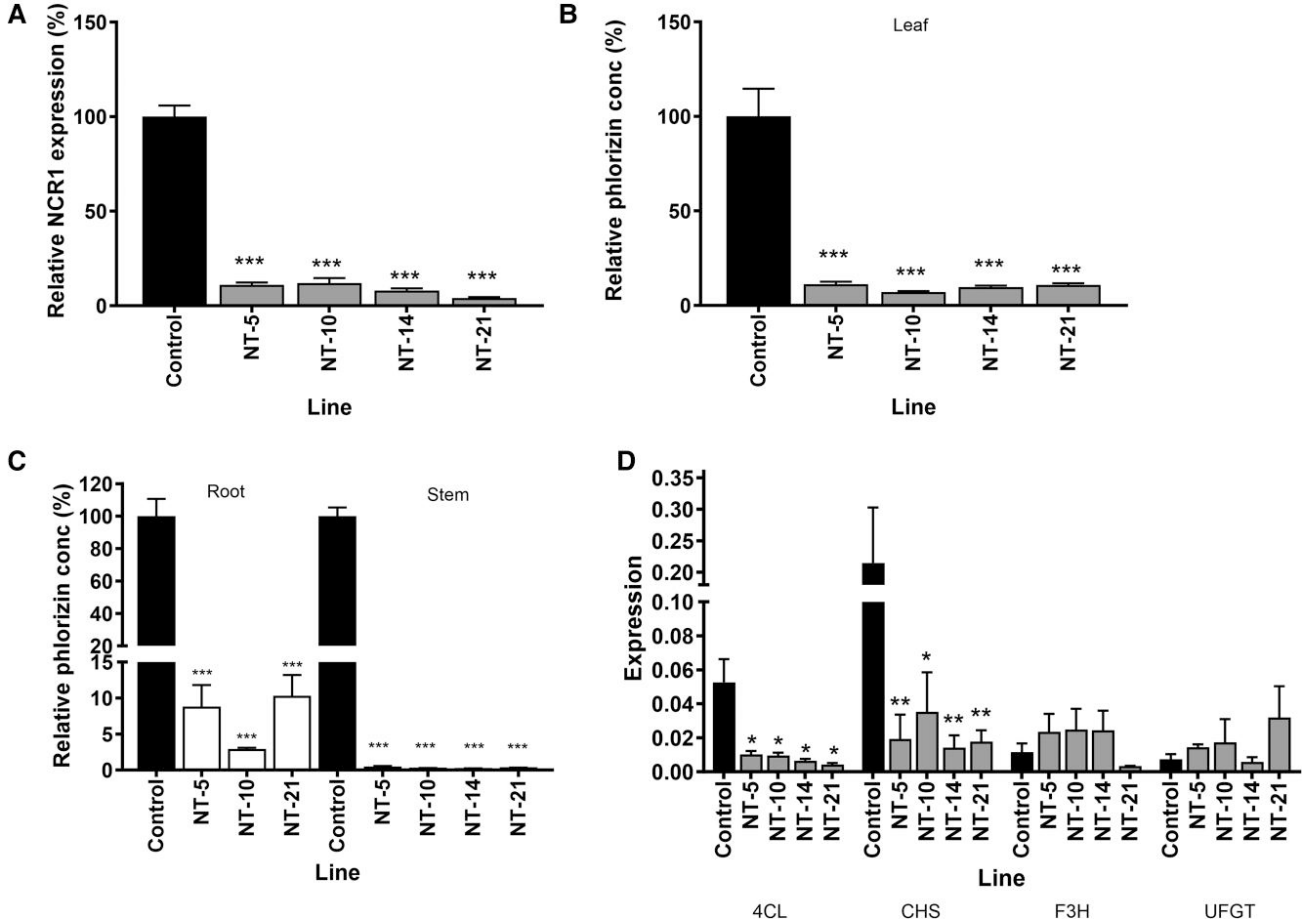
Characterization of transgenic NCR downregulated and control apple lines. **A)** Relative expression of *MdNCR1* in four transgenic NCR (NT) lines was determined by qRT-PCR using RNA extracted from young leaves. Expression was corrected against *MdEF1α* and is given relative to the “Royal Gala” control (value set at 100). Primers and product sizes are given in Supplementary Table S5. Relative concentration of phlorizin in NCR transgenic leaves **B)**, roots and stems **C)** compared to the “Royal Gala” control measured by UPLC. Expression of the control is set at 100%. **D)** Expression of flavonoid pathway related genes in the leaves of four transgenic NCR lines. *Md4CL*, 4-coumaroyl ligase; *MdCHS*, chalcone synthase; *MdF3H*, flavanone 3-hydroxylase and *MdUFGT*, UDP-glucose flavonoid 3-*O*-glucosyltransferase. Data are presented as mean ± SE, *n* = 3 biological replicates. Statistical analysis for **A)**–**D)** was performed in GraphPad Prism: one-way ANOVA using Dunnett's Multiple Comparison Test vs control, *P* < 0.1 = *, *P* < 0.01 = *, *P* < 0.001 = ***.

Relative expression of *MdNCR1* in the four transgenic lines was reduced by >90%, with NT21 showing the maximum reduction at 96% compared with the “Royal Gala” control (Fig. 6A). Foliar concentrations of phlorizin measured by UPLC were significantly reduced by a similar amount (∼90%), with NT10 containing only 7% of the phlorizin concentration found in wildtype (WT) “Royal Gala” (Fig. 6B). No phloretin was detected in WT or the NCR transgenic leaves. Reductions in phlorizin concentrations (90% to 97%) were also observed in root tissue from three transgenic lines grown on their own roots (Fig. 6C, left). Even greater reduction (>99%) in phlorizin concentrations were observed in stem tissue from the NCR transgenics (Fig. 6C, right). The reduced foliar phlorizin concentrations appeared directly related to levels of *MdNCR1* gene expression. The four lines showing the greatest reduction in *MdNCR1* gene expression also showed the greatest reduction in phlorizin concentrations. Lines showing intermediate levels of *MdNCR1* gene repression (Supplementary Fig. S5A) showed intermediate reductions in foliar phlorizin levels (Supplementary Fig. S5B).

The effects of NCR gene downregulation on expression of genes implicated in DHC biosynthesis (*MdPGT1*, *MdHCDBR*, *MdARL1* to *3*, *MdNCR2*) and other genes in the phenylpropanoid/flavonoid pathway (*Md4CL*, *MdCHS*, *MdCHI*, *MdFLS*, *MdF3H*, and *MdUFGT*) were investigated using RT-qPCR. No consistent effect of NCR gene downregulation on the expression of *MdPGT1*, *MdHCDBR*, *MdARL1*, or *MdARL3* expression was observed (Supplementary Fig. S5C). *MdARL2* expression was low in both WT and NCR transgenic leaves. Downregulation of *MdNCR1* and *MdNCR101* did not change *MdNCR2* expression, which remained extremely low and variable. Expression of *Md4CL* and *MdCHS* were consistently downregulated in all lines, suggesting some transcriptional feedback to genes higher in the phenylpropanoid/flavonoid pathway (Fig. 6D). *MdCHI* expression was not detected in WT leaves as reported previously (Dare et al. 2020) and was not detected in the NCR transgenics, too. Expression of *MdF3H* and *MdUFGT* were expressed at similar levels in both WT and NCR transgenic leaves (Fig. 6D). Expression of *MdFLS* was not detected.

LC-MS analysis was used to measure the phenolic compounds present in fully expanded leaves of the four key NCR knockdown lines (Table 1). LC-MS analysis confirmed the reduction of phlorizin in the four transgenic lines and a significant reduction in the accumulation of the DHC di-glycoside phloretin-2ʹ-*O*-xyloglucoside. Concentrations of seven quercetin glycosides increased significantly in all four NCR transgenic lines, with the largest increases found for quercetin 3-*O*-galactoside, quercetin 3-*O*-glucoside and quercetin 3-rutinoside. Concentrations of catechin, epicatechin, and procyanidin C1 and its trimer also increased, while procyanidin B5 and trans-5-*p*-coumaroyl quinic acid decreased. Overall, the decrease in accumulation of DHCs was not compensated by the increase in flavanol-glycosides and other compounds measured, with the four transgenic lines containing only 33% to 39% of the total phenolics extracted from WT “Royal Gala” leaves. The reduction in total phenolics may be associated with the downregulation of *Md4CL* and *MdCHS* observed in the NCR transgenics (Fig. 6D).

**Table 1.** Concentrations of DHCs and other phenolic compounds in the leaves of NCR transgenic lines compared to WT “Royal Gala” controls

Compound	STD/Equ	WT	NT10	NT14	NT21	NT5
DHCs						
XyloPhlz	Phlz	1203 ± 263	107 ± 53	221 ± 29	224 ± 22	190 ± 24
Phlz	STD	78,865 ± 14,580	6104 ± 566	8542 ± 1072	9851 ± 908	9615 ± 868
Total DHC		**80,068** ± **14,843**	**6211** ± **618**	**8763** ± **1099**	**10,075** ± **929**	**9805** ± **891**
% of WT		**100**	**7.8**	**10.9**	**12.6**	**12.2**
Other						
Cat	epiCat	18 ± 2	77 ± 26	79 ± 12	113 ± 45	77 ± 36
CGA	STD	39 ± 0	140 ± 59	86 ± 24	74 ± 16	92 ± 31
epiCat	STD	64 ± 2	201 ± 28	187 ± 8	246 ± 20	251 ± 40
K-rut	STD	10 ± 2	10 ± 2	13 ± 0	9 ± 1	10 ± 1
Nar	STD	2 ± 1	6 ± 2	7 ± 2	8 ± 3	3 ± 2
ProCy B1	STD	4 ± 0	10 ± 1	12 ± 1	11 ± 1	9 ± 2
ProCy B2	ProCy B1	554 ± 251	551 ± 62	461 ± 65	780 ± 169	778 ± 149
ProCy B5	ProCy B1	567 ± 175	174 ± 31	187 ± 25	257 ± 22	204 ± 42
ProCy C1	ProCy B1	1 ± 0	3 ± 0	3 ± 0	5 ± 2	3 ± 1
ProCy trimer	ProCy B1	3 ± 0	8 ± 1	7 ± 1	10 ± 1	10 ± 2
Q-arapy	Q-rut	2412 ± 318	4558 ± 559	4275 ± 253	4705 ± 580	4338 ± 843
Q-gal	Q-rut	984 ± 205	9432 ± 224	8020 ± 714	7608 ± 414	6164 ± 1801
Q-glu	Q-rut	684 ± 48	3722 ± 228	3693 ± 133	5008 ± 1522	2735 ± 499
Q-rha	Q-rut	2080 ± 352	1459 ± 38	1384 ± 299	2173 ± 240	2120 ± 300
Q-rut	STD	10 ± 5	79 ± 14	72 ± 11	49 ± 8	45 ± 11
Q-xyl	Q-rut	864 ± 87	2083 ± 187	2315 ± 180	2275 ± 254	1873 ± 342
t4pCouQA	CGA	554 ± 251	551 ± 62	461 ± 65	780 ± 169	778 ± 149
t5pCouQA	CGA	567 ± 175	174 ± 31	187 ± 25	257 ± 22	204 ± 42
Total other		**8622** ± **293**	**23,240** ± **1153**	**21,448** ± **1293**	**24,368** ± **2593**	**19,646** ± **4199**
Fold change		**1**	**2.7**	**2.5**	**2.8**	**2.3**
Total DHC + other		**88,690** ± **14,551**	**29,451** ± **1764**	**30,211** ± **2256**	**34,443** ± **3238**	**29,500** ± **4699**
% of WT		**100**	**33.2**	**34.1**	**38.8**	**33.2**

Phenolic compounds were extracted from the leaves of four NCR transgenic lines (NT5, 10, 14, and 21) and WT and analyzed by LC-MS. DHC, dihydrochalcone; xyloPhlz, phloretin-2′-*O*-xyloglucoside; Phlz, phlorizin; Cat, catechin; CGA, chlorogenic acid; epiCat, epicatechin; K-rut, kaempferol 3-*O*-rutinoside; Nar, naringenin; Procy, procyanidin; Q-arapy, quercetin 3-*O*-arabinopyranoside; Q-gal, quercetin 3-*O*-galactoside; Q-glu, quercetin 3-*O*-glucoside; Q-rha, quercetin 3-*O*-rhamnoside; Q-rut, quercetin 3-rutinoside; Q-xyl, quercetin 3-*O*-xyloside; t4pCouQA, trans-4-*p*-coumaroyl quinic acid; t5pCouQA, trans-5-*p*-coumaroyl quinic acid. Compounds given in bold are 3-fold higher/lower compared to WT. All concentrations in µg·g^−1^ fresh weight. Concentrations were measured against authentic standards (STD), or equivalence (equ) as indicated. Data are mean ± SE (*n* = 3 biological replicates).

### In situ localization of apple NCRs

**Figure 7.**
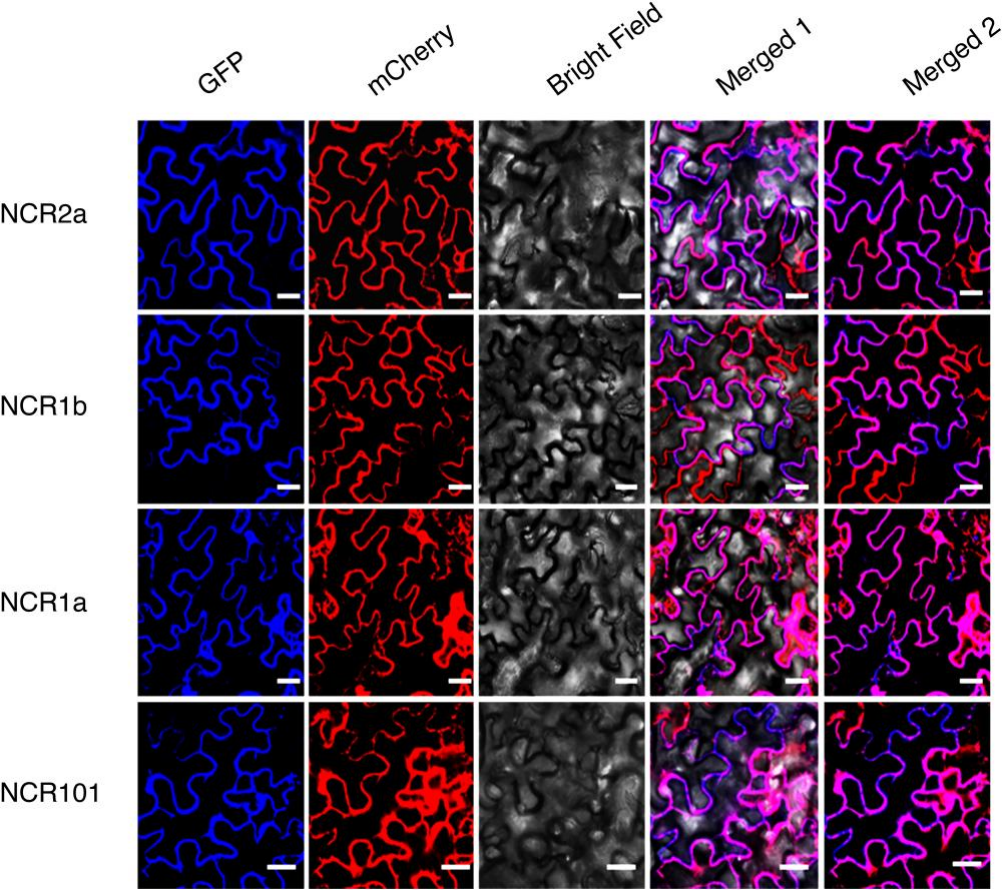
Subcellular localization of NCR proteins in *N. benthamiana*. C-terminal translational fusion constructs of GFP to MdNCR2a, MdNCR1a, MdNCR1b, and MdNCR101 were transiently expressed in *N. benthamiana* leaves and analyzed with a confocal laser-scanning microscope. GFP, GFP fluorescence; mCherry, RFP fluorescence (from the VAC-RK vector localized to the vacuolar membrane), Brightfield, light microscopy images; Merged 1 GFP, RFP, and Brightfield images merged; Merged 2, GFP, and RFP fluorescence images merged. Scale bars, 25 µm.

TargetP predictions suggest that NCR proteins are not targeted to the chloroplast via a canonical N-terminal targeting peptide (Supplementary Table S4B) as previously observed for some QRs, e.g. CsChlAOR (Yamauchi et al. 2011). However, AtceQORH, which is also predicted to have no chloroplast targeting peptide, was shown to be localized to the inner membrane of the chloroplast envelope via a cryptic internal targeting domain (Miras et al. 2002). NCR genes share 63% to 78% deduced amino acid identity in this internal targeting domain (Supplementary Table S4C). Therefore, to determine the subcellular localization of apple NCR proteins, full length ORFs of *MdNCR1a*, *1b*, *MdNCR101*, and *MdNCR2a* were each fused at the C-terminus to GFP and infiltrated into the leaves of *N. benthamiana*. Expression of GFP was then analyzed by confocal laser scanning microscopy (Fig. 7).

No GFP signals were found that were associated with chloroplasts or that could be overlaid with chlorophyll autofluorescence, indicating the NCR proteins are not localized to chloroplasts by a canonical targeting sequence or cryptic chloroplast targeting domain. Strong GFP fluorescence signals of similar intensity were observed for all NCR proteins that were localized to the vacuolar membrane (Fig. 7, column 1). These signals merged with strong red RFP fluorescence signals (Fig. 7, column 2) for the vacuolar membrane marker in the control VAC-RK (mCherry) vector. The merged signal is visualized as the predominant pink color in Fig. 7, columns 4 and 5. No GFP or RFP fluorescence was detected in uninfiltrated tissues. For the positive control 35S-GFP construct, signals were found throughout the cell (Supplementary Fig. S6). These results indicate that NCR proteins are likely to be located at the vacuolar membrane with other proteins involved in phenylpropanoid biosynthesis (Hrazdina and Jensen 1992).

### Modeling of apple NCR genes

The structures of MdNCR1a–c, MdNCR101, and MdNCR2a were modeled using AlphaFold, with very high confidence over their entire length. A Foldseek (van Kempen et al. 2024) search against the PDB identified AtceQORH as the closest structural match with MdNCR1a–c. The proteins share 70.8% sequence identity (Supplementary Fig. S7) and an rmsd of 0.38Å over 302 C-alpha atoms. Structural comparison (Supplementary Fig. S8) showed that, like AtceQORH, MdNCR1a–c have a very large substrate binding pocket, compatible with its ability to accept bulky compounds like naringenin chalcone as substrate. This contrasts with the structure of the more distantly related strawberry oxidoreductase FaEO (PDB code 4IDE), which has a much smaller substrate binding pocket to accept the small 4-hydroxy-5-methyl-2-methylene-3(2H)-furanone as substrate (Schiefner et al. 2013). Indeed, a variation in the length of the loops capping their respective substrate-binding pockets (residues 96 to 104 in MdNCR1a and AtceQORH; residues 103 to 114 in FaEO) creates a much more spatially restrained substrate-binding site for FaEO, compared with MdNCR1a–c (Supplementary Fig. S8).

**Figure 8.**
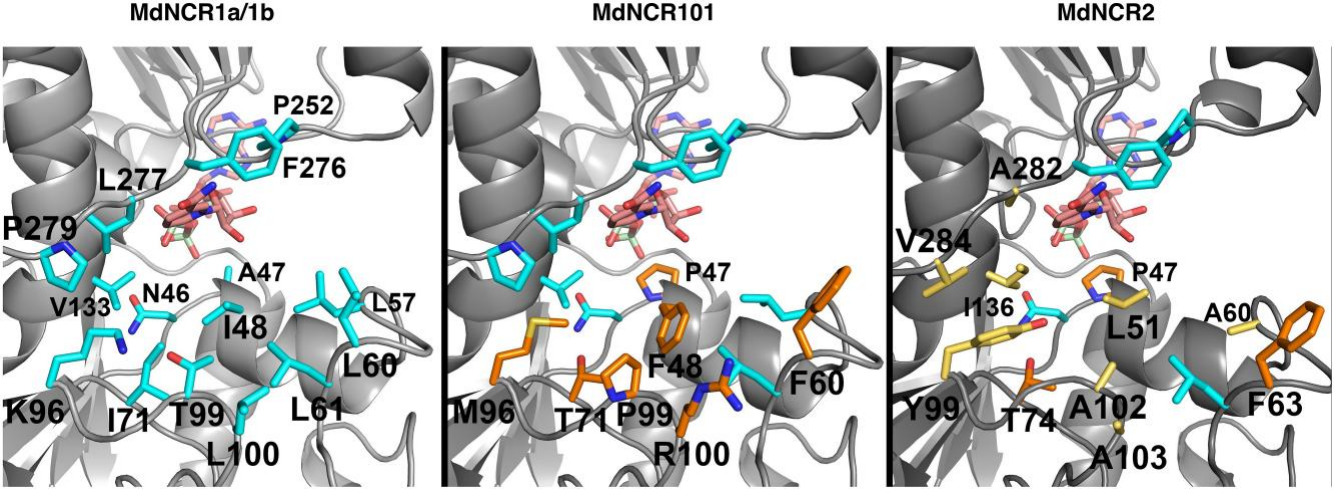
Modeling of NCR proteins using AlphaFold. Substrate binding pockets of the MdNCR1a–c (left), MdNCR101 (middle) and MdNCR2 (right) models. The 15 amino acids shaping the substrate binding pocket of NCR1a–c are shown in stick mode with carbon atoms in cyan and labeled (left). The seven amino acids differences between the MdNCR101 and the MdNCR1a–c substrate binding pockets are shown in stick mode with carbon atoms in orange (middle), while the 11 amino acid differences observed between the MdNCR2a and the MdNCR1a–c, substrate binding pockets are shown in stick mode with carbon atoms in yellow if unique to MdNCR2a or orange if shared with MdNCR101 (right). The superimposed NAPD moiety is shown in stick mode, with carbon atoms in pink and phosphate atoms in pale green, respectively. Nitrogen, oxygen, and sulfur atoms are in blue, red, and amber, respectively.

Sequence alignments between MdNCR1a–c, MdNCR101, and MdNCR2a show that most of their amino acid differences are located in their substrate binding domains (residues 1 to 132, Supplementary Fig. S7). When mapped onto their predicted structures, none of the amino acid differences observed between MdNCR1a, MdNCR1b, and MdNCR1c were located within their substrate binding pockets (Fig. 8), in agreement with the ability of these three enzymes to efficiently reduce the same substrate, naringenin chalcone. In contrast, nearly half of the fifteen amino acids of the substrate-binding pocket of MdNCR101 (7/15) and ∼3/4 (11/15) of that of MdNCR2a varied when compared with MdNCR1a–b (Fig. 8). Hence, both MdNCR101 and MdNCR2a are predicted to present either shape and/or physico-chemical properties in their substrate binding pockets that differ from those of MdNCR1a–c, providing a rationale for the difference in activity observed in transient in planta assays between these closely related enzymes.

## Discussion

MdNCR1 fulfills the requirements to be the missing enzyme required for DHC biosynthesis in apple leaves. Phylogenetic analysis indicates that the NCR1 clade is specific to *Malus*, which accumulate high concentrations of DHCs. In contrast, the clade is not present in *Pyrus* and related members of the Maleae, which do not accumulate DHCs. *MdNCR1* genes are highly expressed in apple leaves, roots, stems and young fruit where phlorizin accumulates to high concentrations, and are not expressed in mature and ripening fruit tissues where accumulation is much lower. The pattern of *MdNCR1* expression also mirrors the pattern for NCR enzyme activity previously reported by Dare et al. (2020). Recombinant protein, kinetic and transient expression analyses indicate that MdNCR1 enzymes can catalyze efficient production of phloretin both in vitro and in planta. Finally, transgenic apple plants in which *MdNCR* genes were downregulated by RNAi, show greatly reduced accumulation of DHCs in all tissues studied.

The NCR1 clade specific for DHC production in *Malus* is associated with gene duplication and neofunctionalization of NCR genes that are present in *Pyrus* and other Rosaceae species. Other genes involved in DHC synthesis (*MdPGT1*), and catabolism (polyphenol oxidase, *PPO05* and *PPO06*; B-glucosidase, *BGLU13*), have also been reported to have undergone similar duplication and neofunctionalization (Elejalde-Palmett et al. 2019; Zhao et al. 2024). The enhanced capacity for DHC biosynthesis and catabolism is likely linked to improved apple resistance to pests and diseases such as two spotted mites, *Valsa* canker and powdery mildew (Zhou et al. 2019; Dare et al. 2020; Zhao et al. 2024). Although *MdNCR2a* did not produce DHCs when transiently expressed in *N. benthamiana*, the recombinant protein (if soluble and active) might allow identification of potential substrates for the broader NCR-related enzyme family. Transgenic downregulation of NCR-related genes in *Pyrus* and other Rosaceae would allow confirmation of their substrate preference as double bond reductases in planta. Although high concentrations of DHCs are typically associated with *Malus* leaves and fruit, other plants e.g. as *Lithocarpus* spp. (Qin and Liu 2003; Sun et al. 2015) also accumulate DHCs in leaves. It will be of interest to see if the pathway to DHC production in other species occurs primarily via direct reduction of naringenin chalcone or via reduction of *p*-coumaroyl CoA.

*MdNCR1* gene expression is undetectable in mature and ripening fruit, yet DHC production (mainly phlorizin and phloretin xyloglucoside) occurs in these tissues (Gosch et al. 2010). MdNCR1 may account for the DHCs observed in mature/ripe fruit if DHCs are synthesized in young fruit tissues and stored before maturity. Alternatively, the *MdNCR101* gene may account for DHC production in fruit, because it is expressed at low levels in fruit tissue and shows weak NCR activity. A further possibility is that *MdHCDBR* and other *MdARL* genes that are expressed in fruit tissue may be important for DHC production in fruit via the reduction of *p*-coumaroyl CoA to form dihydro-*p*-coumaroyl CoA. This question may be resolved in 2 to 3 years' time, when the transgenic NCR and *MdHCDBR/MdARL2* apple plants set fruit.

The downregulation of *MdHCDBR* and *MdARL2* by RNAi in transgenic apple leaves also downregulated two further related genes *MdARL1* and *MdARL3* (Supplementary Fig. S2). From this experiment we concluded that none of these four genes are responsible for the high concentrations of DHCs observed in apple leaves. Recombinant MdHCDBR expressed in *E. coli* can reduce *p*-coumaroyl CoA to dihydro-*p*-coumaroyl CoA (Ibdah et al. 2014), however no activity toward *p*-coumaroyl CoA was detected when the enzyme was expressed in yeast (Eichenberger et al. 2017; Caliandro et al. 2021). Three substrates for MdHCDBR expressed in yeast were identified, phenyl-3-buten-2-one, 4-hydroxybenzalacetone, and *p*-coumaroyl aldehyde (Caliandro et al. 2021). Whether *MdHCDBR* and *MdARL1–3* function to reduce these three substrates in planta is unclear. However, the availability of the transgenic Hn2 knockdown lines may allow target compounds and products to be identified in apple leaves and later in fruit.

Enzymes in the phenylpropanoid pathway may form part of a membrane-associated enzyme complex (Winkel-Shirley 1999), while most conjugated flavonoids including glycosides, are found primarily in the vacuole (Zhao and Dixon 2010). It has been suggested that pathway intermediates are channeled between the individual enzymes and not released into the cytosolic environment (Hrazdina and Jensen 1992). Localization of MdNCR1 proteins to the vacuolar membrane and the acidic pH optima for MdNCR1b support NCR enzymes having a role in the flavonoid pathway and production of DHC glycosides that are located in the vacuole. The NCR enzyme may associate with MdCHS to channel the unstable naringenin chalcone substrate and with MdPGT1 to glycosylate the highly reactive phloretin product, as neither of these compounds is readily detected in apple leaf extracts. Precedence for similar types of interaction is provided by CHI-like proteins that can bind CHS to rectify enzyme promiscuity (Waki et al. 2020). Naringenin chalcone itself stabilizes this CHS-CHI interaction to create a positive feedback loop while naringenin destabilizes it (Wolf-Saxon et al. 2023).

The affinity of MdNCR1b for naringenin chalcone is similar to that reported for the major substrates of AtceQORH (medium-chain (C ≥ 9) to long-chain (18 carbon atoms) compounds derived from poly-unsaturated fatty acid peroxides), although the catalytic efficiency is lower (Curien et al. 2016). However, the reduced catalytic efficiency of MdNCR1b may not reflect the true physiological rate. To minimize naringenin chalcone isomerization to naringenin, reactions were carried at pH 3.0, which is lower than the pH typically found in plant vacuoles (pH 5.0) or would be found if the protein was part of a membrane-associated enzyme complex. The NCR enzyme needs to be competitive with MdCHI for the naringenin chalcone substrate. The affinity of CHI enzymes for naringenin chalcone is reported to be in the low μM range (e.g. Park et al. 2018, 2021), similar to that for MdNCR1b. In apple, *MdCHI* expression is low in leaves (Dare et al. 2020), while our data indicate that *MdNCR1a–c* expression is high (Fig. 5A). This suggests the accumulation of DHCs results from a combination of high NCR expression and low CHI expression in apple rather than a difference in protein activity.

In earlier studies focused on altering phlorizin concentrations by downregulation of *MdCHS* and *MdPGT1*, it was reported that transgenic plants showed a severely stunted growth habit associated with a reduction in phlorizin accumulation of up to 90% (Dare et al. 2013, 2017; Zhou et al. 2019). In contrast, similar reductions in phlorizin content by heterologous expression of *AtCHI* (Dare et al. 2020) and by downregulation of *MdNCR* reported here resulted in plants with no obvious changes in growth habit. CHIox and NCR transgenic leaves both show elevated concentrations of flavonols and flavan-3-ols, presumably because of an increased flux to the lower flavonoid pathway, whereas the concentrations of these compounds are reduced in *MdCHS* transgenics. How these compounds affect plant growth is unclear, but studies in model systems suggest an involvement in auxin transport (Jacobs and Rubery 1988; Yin et al. 2014; Ng et al. 2015).

Identifying the missing enzyme required for DHC production provides opportunities for manipulation of its content in apple and other fruit. DHCs have a broad range of biological activities that can benefit human health (Korbin 2023). Phlorizin has been the most intensively studied DHC because of its effects on glucose absorption and excretion, and for its potential use in the treatment of type 2 diabetes (Ehrenkranz et al. 2005). Understanding the full pathway to DHC production in plants should accelerate the development of new cultivars with improved health properties. Identification of NCR may also allow more efficient biosynthesis of DHCs by biotechnological approaches such as biopharming and biofermentation in yeast. Such approaches have already been applied to other enzymes required for the production of DHCs (Eichenberger et al. 2017; Yang et al. 2024). Metabolic engineering of NCR may allow large quantities of trilobatin to be produced for use as a natural sweetener or other DHCs to be produced as building blocks for drug biosynthesis.

## Materials and methods

### RNA extraction and cloning

RNA was extracted from three biological replicates of each tissue using the Spectrum Plant Total RNA kit (Sigma-Aldrich, St Louis, MO, USA) and DNAse I treated using a DNA-*free* Kit (Invitrogen, Carlsbad, CA, USA). The quantity and quality of extracted RNA were assessed with a Nanodrop 2000 spectrophotometer (Thermo Scientific, https://www.thermofisher.com). For gene cloning and RT-qPCR, cDNA was synthesized from 1 µg of total RNA in a total volume of 20 µL using Qiagen reverse transcriptase according to the manufacturer's instructions (http://www.qiagen.com).

### Transient expression in *N. benthamiana* leaves

Primers designed to *MdNCR1/101* and *MdNCR2/201* were used to amplify full length ORFs from apple leaf cDNA using the primers listed in Supplementary Table S5. Genes were cloned into the pDONR221 vector (Invitrogen), then transferred into the binary vector pHEX2 (CaMV 35S:ORF:nos-3ʹ). All constructs were electroporated into *Agrobacterium tumefaciens* strain GV3101. Transient expression in *N. benthamiana* was performed as described in Green et al. (2012). Leaves infiltrated with pHEX2_GUS construct (Nieuwenhuizen et al. 2009) were used as negative controls and leaves infiltrated with pHEX2_HCDBR (Wang et al. 2020) as positive controls. Construction of pHEX2_MYB10 was described previously (Espley et al. 2007). Each test construct was co-infiltrated in a 1:1:1:1:1 ratio with pHEX2_PGT1 to glycosylate phloretin to phlorizin, pHEX2_CHS, pHEX2_MYB10 to increase flux through the flavonoid pathway, and pBIN19 as a repressor of gene silencing. Leaves were collected after 7 d and 100 to 300 mg of tissue was ground with liquid nitrogen for metabolite extraction.

### Metabolite extraction for HPLC, UPLC, and LC-MS analysis

Phenolics for HPLC analysis were extracted from leaf tissue (100 to 300 mg) in five volumes of 100% methanol containing 0.1% HCl for 2 h at room temperature (RT). Extracts were then centrifuged for 5 min at 5,000 × *g* at RT to pellet the cell debris. A 500 μL aliquot was removed and dried down in a vacuum evaporator at 30 °C. Samples were then resuspended in 500 μL of 20% methanol and filtered through a 0.45 μm nylon syringe filter (https://www.dksh.com). HPLC-diode array detector (DAD) analysis was carried out using a Dionex Ultimate 3000 system (Sunnyvale, CA) equipped with a DAD as described by André et al. (2012).

Phenolics for UPLC analysis were extracted from leaf, root, stems, and fruit tissues (100 to 300 mg) in five volumes of 80% methanol + 1% formic acid for 2 h at RT. Extracts were then centrifuged, pelleted, and filtered as described for HPLC analysis. Analysis was carried out on an Acquity UPLC system (Waters, Milford, MA, USA) equipped with a photodiode array detector. An aliquot of 5 μL was injected onto an Acquity UPLC HSS T3 column (2.1 × 100 mm, 1.8 μm particle size (Waters) at 50 °C and a flow rate of 0.65 mL·min^−1^. The eluents were 0.1% (v/v) formic acid in water (A) and 0.1% formic acid in acetonitrile (B). The gradient was as follows: 0 min, 15% B; 2 min, 15% B; 10 min, 50% B; 12 min, 50% B; 17 min, 95% B; 19 min, 95% B; 19.5 min, 5% B; 22 min, 15% B. Compounds were detected at 280 nm according to absorption maxima, compared with authentic standards and quantified using a five-point calibration curve.

Phenolics for LC-MS were extracted from leaf tissue in ten volumes of 80% ethanol + 2% formic acid and analyzed as described previously (Kumar et al. 2022).

### Recombinant expression in *E. coli*

The MdNCR1b ORF in pDONR221 was transferred into the pET300 vector according to the manufacturer's instructions (Invitrogen). The construct was transformed into BL21 DE3 codon plus (RIL) cells, and recombinant protein was expressed in *E. coli* and purified by FPLC as described previously (Green et al. 2007). Protein was eluted with a 0.5 to 300 mm imidazole gradient. Protein concentration was calculated by NanoDrop 2000 at A280 nm. Purity was assessed on SDS-PAGE gels (Supplementary Fig. S4A).

### Enzyme activity assays

MdNCR1b activity assays with naringenin chalcone were performed in triplicate in 200 μL reactions using ∼250 ng of purified recombinant protein. Reactions were performed in Bis-Tris Propane buffer (0.1 m) with 100 μM naringenin chalcone and 2 mm NADPH at pH 3.0. Reactions were performed at 30 °C for 30 min and terminated by addition of 200 μL of acetonitrile/formic acid (99:1 *v/v*) then filtered through a 0.45 μM syringe filter (https://www.dksh.com). Boiled enzyme and pET300 vector controls were run in parallel with all enzyme reactions. Reactions were shown to be linear with respect to time and enzyme concentration under standard conditions. The apparent Km value for naringenin chalcone was determined by varying the naringenin chalcone concentration from 200 to 0.16 μM with a fixed NADPH concentration of 400 μM. The Km value for NADPH was determined by varying the concentration of NADPH from 800 to 0.2 μM with a fixed naringenin chalcone concentration of 100 μM.

MdNCR1b reactions with other phenolic substrates were performed in 200 μL reactions at 30 °C for 16 h using 10 μg of protein at pH 6.5 with 1 mm substrate and 2 mm NADPH. MdNCR1b reactions with volatile substrates were performed in 700 μL reactions using 10 μg of protein at pH 3.0 with 2 mm substrate and 0.4 mm NADPH. Reactions were incubated at 30 °C overlaid with 300 μL of pentane:diethyl ether (1:1). After 16 h reactions were vortexed, then frozen for 4 h. The solvent layer was removed for GC-MS analysis. MdHCDBR and MdNCR1b reactions with *p*-coumaroyl CoA were performed using conditions described previously (Ibdah et al. 2014). Reactions (100 μL) were incubated at 30 °C for 16 h using 10 μg of purified recombinant protein at pH 5.0 in citrate/phosphate buffer (0.2 m) with 180 μM *p*-coumaroyl CoA and 2.5 mm NADPH. MdHCDBR reactions with naringenin chalcone were performed as described for MdNCR1b but using 25 μg of purified recombinant protein and at pH 6.5.

*p*-Coumaroyl CoA was synthesized as described by Dare et al. (2020). Naringenin chalcone and all other chemicals were purchased from Sigma Aldrich.

LC-MS analysis of NCR reaction products employed an LTQ linear ion trap mass spectrometer fitted with an ESI interface (ThermoFisher Scientific) coupled to an Ultimate 3000 UHPLC instrument (ThermoFisher Scientific). Phenolic compound separation was achieved using a Hypersil Gold aQ 1.9 µm (ThermoFisher Scientific), 150 × 2 mm analytical column maintained at 45 °C. Solvents were (A) acetonitrile + 0.1% formic acid and (B) water + 0.1% formic acid and the flow rate was 200 µL·min^−1^. The initial mobile phase, 5% A/95% B, was held for 5 min then ramped linearly to 10% A at 10 min, 17% A at 25 min, 23% A at 30 min, 30% A at 40 min, 97% A between 48 and 53 min before resetting to the original conditions. Sample injection volume was 2 µL. MS data were acquired in the negative mode using a data-dependent LC-MS^3^ method. This method isolates and fragments the most intense parent ion to give MS^2^ data, then isolates and fragments the most intense daughter ion (MS^3^ data). The ESI voltage, capillary temperature, sheath gas pressure, and sweep gas were set at −10 V, 275 °C, 40, and 5 psi, respectively.

Separation of CoA products separation was achieved using a Hypersil Gold aQ 1.9 µm (ThermoFisher Scientific), 150 × 2 mm analytical column maintained at 30 °C. Solvents were (A) methanol and (B) 0.1% ammonium acetate in water and the flow rate was 200 µL·min^−1^. The initial mobile phase, 5% A/95% B, was ramped linearly to 10% A at 10 min, 60% A at 15 min, 90% A at 20 min, before holding for 5 min and resetting to the original conditions. Sample injection volume was 2 µL. MS data were acquired in the positive mode using a data-dependent LC-MS^3^ method. The ESI voltage, capillary temperature, sheath gas pressure, and sweep gas were set at 9 V, 275 °C, 40, and 5 psi, respectively.

Volatile NCR reaction products were analyzed by GC-MS on a Pegasus BT GC-TOF-MS mass spectrometer (LECO Corporation, St. Joseph, MI, USA) coupled to an Agilent 7890 gas chromatograph (Agilent Technologies, Santa Clara, CA, USA). Solvent volatile collection and GC-MS analysis were performed according to previously described methods (Zeng et al. 2020; Wang et al. 2023), with some modifications as described in Supplementary Method S1.

### RT-qPCR reaction

RT-qPCR was performed on a LightCycler 480 platform using the LightCycler 480 SYBR Green master mix in a reaction volume of 5 µL. At least three technical replicates were used, and cDNA was replaced by water to test for false amplification. Data were analyzed using the Target/Reference ratio calculated with the LightCycler software 1.5 with PCR efficiency corrections for each primer pair (Roche). The analysis was performed relative to the apple reference gene *MdEF1*α. Cycling conditions were: 5 min at 95 °C; 40 cycles of 10 s at 95 °C, 10 s at 60 °C, and 20 s at 72 °C; followed by melting curve analysis: 95 °C for 5 s, 65 °C for 60 s then ramping at 0.10 °C·s^−1^ to 97 °C. Gene-specific primers, product sizes and primer efficiencies are displayed in Supplementary Table S5.

### Generation and growth of transgenic plants

Two 300 bp sequences extracted from *MdHCDBR* (nucleotides 191 to 490) and *MdARL2* (191 to 490 bp) were concatenated and flanked by the Gateway attL1 and attL2 sequences in Geneious Prime (Version 2022.0.1). Similarly, three 200 bp fragments extracted from *MdNCR1a*, *MdNCR1b*, and *MdNCR101* (each from nucleotides 200 to 399) were concatenated and flanked by the Gateway attL1 and attL2 sequences in Geneious Prime. Each 600 bp fragment was synthesized by GenScript (www.genscript.com) and cloned into the pUC57 vector. RNAi hairpin constructs were produced using LR reactions into the binary vector pTKO2 vector following manufacturer's instructions (Invitrogen). pTKO2 utilizes the CaMV 35S promoter and the transcriptional terminator of the *Arabidopsis ACT2* gene (Snowden et al. 2005).

Binary vectors were electroporated into *A. tumefaciens* strain LBA4404 and used to generate multiple independent transgenic *M.* × *domestica* (Borkh.) “Royal Gala” apple plants (Yao et al. 1995). Transgenic plants were micro-grafted onto “M9” rootstocks and grown in a containment glasshouse alongside untransformed grafted “Royal Gala” WT controls. Transgenic NCR and MdHCDBR/MdARL2 plants were grown under ambient conditions (temperature min 18 °C/max 30 °C night/day, 14 h/10 h light/dark in summer) and fed daily via an automatic fertigation system with Wuxal Super. Transgenic MdMYB10, MdPGT1, CHIox and MdCHS apple lines were grown under the same conditions.

Transgenic NCR plants were multiplied on maintenance medium (Yao et al. 2013) containing timentin (300 mg·L^−1^) but no kanamycin. When shoots were 2 cm in length they were transferred to the rooting medium described in Yao et al. (2013), but with 15 g·L^−1^ sucrose and 0.5 mg·L^−1^ IBA. Rooting media also contained timentin (300 mg·L^−1^) but no kanamycin. After 6 weeks, roots became visible and plantlets were transferred to the greenhouse.

### Localization

Full-length NCR ORFs were amplified without a stop codon (using primers in Supplementary Table S5) and inserted into pCAMBIA2300-GFP (Leclercq et al. 2015). The control vector VAC-RK was used to localize RFP fluorescence to the vacuolar membrane (Shaanxi AUG Biotechnology Co. Ltd.). Each translational fusion construct was infiltrated into *N. benthamiana* leaves at optical density at 600 nm = 0.2. After 3 d incubation, fluorescence images were acquired using a confocal laser-scanning microscope (FV3000, Olympus, Tokyo, Japan) with a 40 × 0.95 numerical aperture objective (UPLSAPO 40×, Olympus). For imaging, GFP and RFP were excited using an Argon laser at 488 and 552 nm wavelengths, respectively. The emission filters were 505 to 550 nm for GFP, and 590 to 640 nm for RFP. Chlorophyll autofluorescence was monitored using 488 nm excitation wavelengths and 650 to 750 nm detection windows. Measurements were controlled with the manufacturer's FV31S-SW (Olympus) software. All fluorescence experiments were repeated independently at least three times.

### Modeling

Protein models for MdNCR1a–c, MdNCR101, and MdNCR2a were built using LocalColabFold (running ColabFold 1.5.2 (Mirdita et al. 2022) and AlphaFold 2.3.1 (Jumper et al. 2021)). NADP from the AtceQORH crystal structure (PDB entry 5A4D) was superimposed to the models using AlphaFill (Hekkelman et al. 2023). Models were visualized, superimposed and analyzed using PyMOL 2.5.7 (The PyMOL Molecular Graphics System, Version 2.0 Schrödinger, LLC).

### Accession numbers

Sequence data from this article can be found in the GenBank/EMBL data libraries under accession numbers *MdNCR1a* (PP495130), *MdNCR1b* (PP495131), *MdNCR1c* (PQ045267), *MdNCR101* (PP495132), *MdNCR2a* (PP495133), *PcNCR101* (PP495134).

## Supplementary Material

kiae515_Supplementary_Data

## Data Availability

The data underlying this article are available in the article and in its online supplementary material.

## Supplementary data

**Supplementary Figure S1.** Phylogram of MdHCDBR and alkenal reductase like (ARL) gene models from apple and other species.

**Supplementary Figure S2.** Rapid screening of *MdHCDBR* and *MdARL2* transgenic lines.

**Supplementary Figure S3.** NCR alignment, phylogeny and additional UPLC trace.

**Supplementary Figure S4.** Purification of recombinant MdNCR1 and optimizing NCR reaction conditions.

**Supplementary Figure S5.** Screening of transgenic NCR lines by qPCR and HPLC.

**Supplementary Figure S6.** Subcellular localization of 35S-GFP in *N. benthamiana*.

**Supplementary Figure S7.** Sequence alignment between MdNCR1a–c, MdNCR101 and MdNCR2a.

**Supplementary Figure S8.** Structural comparisons between the MdNCR1a–c, AtceQORH and FaEO.

**Supplementary Method S1.** NCR product analysis by GC-MS analysis.

**Supplementary Table S1.** Expression and amino acid identities of apple MdHCDBR and MdARL genes.

**Supplementary Table S2.** Identification of NCR gene candidates.

**Supplementary Table S3.** Genomic location, open reading frame size and gene annotation of each NCR gene model.

**Supplementary Table S4.** Amino acid identities and targeting predictions for apple NCR genes.

**Supplementary Table S5.** Oligonucleotide primers used for RT-qPCR and cloning.

## Dive Curated Terms

The following phenotypic, genotypic, and functional terms are of significance to the work described in this paper:

BGLU13 Gramene: AT5G44640
BGLU13 Araport: AT5G44640

NADPH CHEBI: CHEBI:16474

naringenin CHEBI: CHEBI:50202

CGA Gramene: Chlorogenic acid
CGA Araport: Chlorogenic acid

FLS Gramene: AT5G08640
FLS Araport: AT5G08640

CHS Gramene: AT5G13930
CHS Araport: AT5G13930
