# Supplementary material for: Naringenin chalcone carbon double-bond reductases mediate dihydrochalcone biosynthesis in apple leaves
Source: Plant Physiol. 2024 Sep 29;196(4):2768–83. doi: 10.1093/plphys/kiae515 (PMC11638483; doi:10.1093/plphys/kiae515)
Supplement: kiae515_Supplementary_Data [file kiae515_supplementary_data.zip › PP2024RA00532R2_Supplemental_Data.pdf]

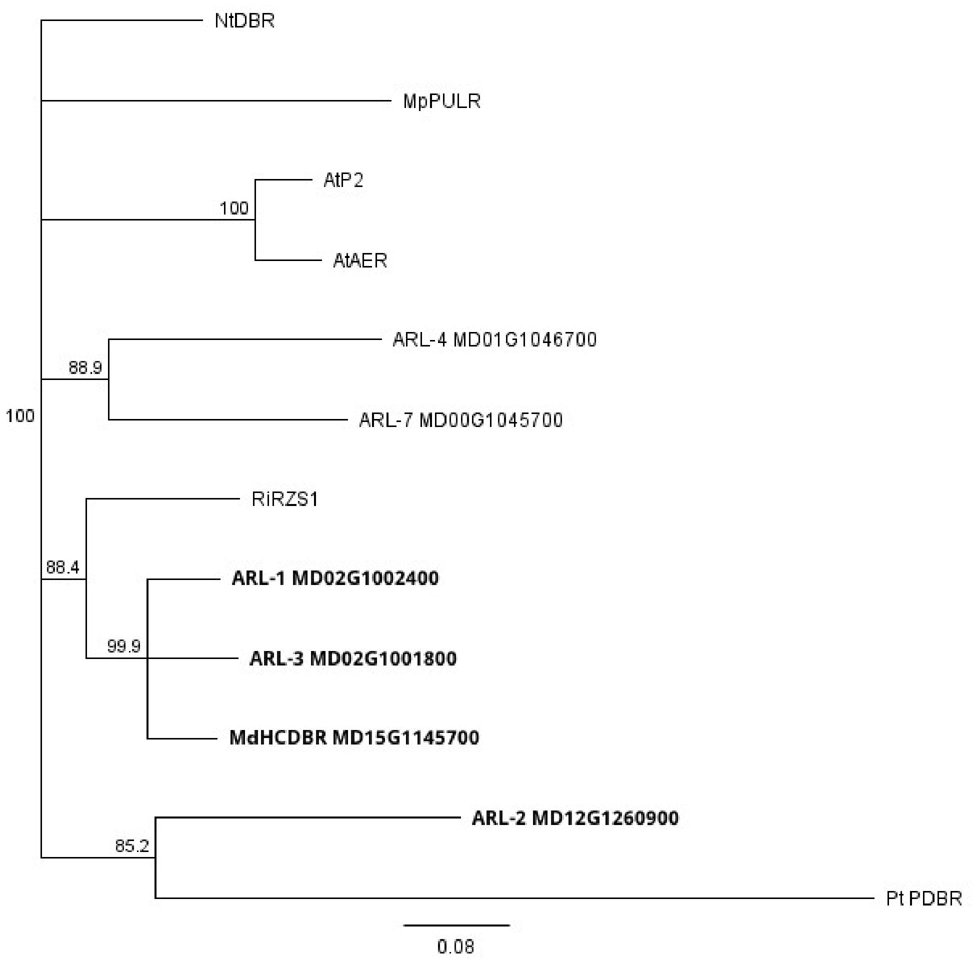

Supplementary Figure S1. Phylogram of MdHCDBR and alkenal reductase like (ARL) gene models from apple and other species. Amino acid alignments were generated with Clustal Omega in Geneious Prime (Version 2022.0.1). Trees were inferred using the Maximum Likelihood method based on the JTT matrix-based model (Jones et al., 1992, Comput Appl Biosci **8:** 275-282). Percentage bootstrap values >80% (1000 replicates) are shown. Branch lengths measure the number of substitutions per site relative to the scale bar. Apple gene models were obtained from Genome Database for Rosaceae (Jung et al., 2019; Nucleic Acids Res **47:** D1137-D1145). Genes in bold were targeted for downregulation in the RNAi construct. *NtDBR,* (Q9SLN8.1), *Nicotiana tabacum* 2-alkenal reductase*; MpPULR* (Q6WAU0.1), *Mentha piperita* pulegone reductase; *AtP2* (Q39173.2), *Arabidopsis thaliana* NADP-dependent alkenal double-bond reductase P2*; AtAER* (Q39172.1), *Arabidopsis thaliana* NADP-dependent alkenal double-bond reductase P1*; RiRZS1* (AEL78825.1), *Rubus idaeus* ketone/zingerone synthase 1*, PtPPDBR* (ABG91753.1), *Pinus taeda* phenylpropenal double-bond reductase.

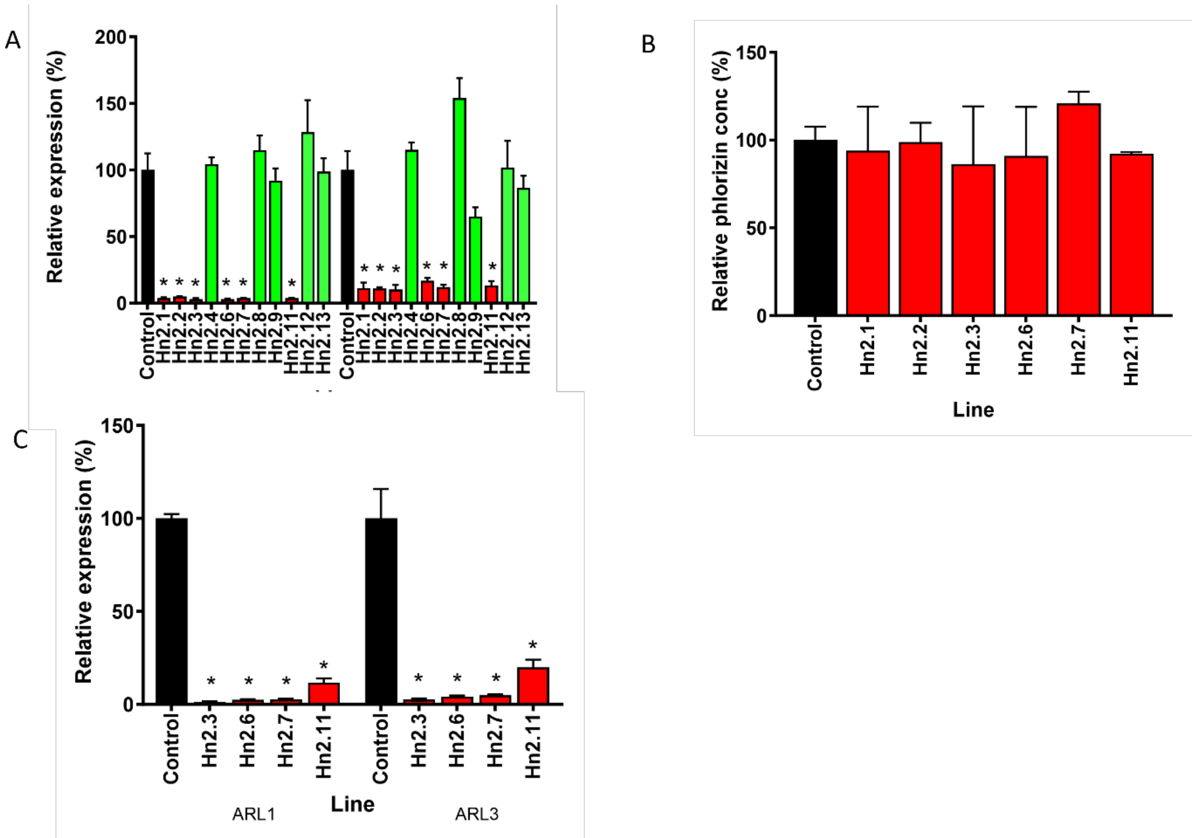

Supplementary Figure S2. Rapid screening of *MdHCDBR* and *MdARL2* transgenic lines. (A) Relative expression of *MdHCDBR* and *MdARL2* in the leaves of thirteen transgenic MdHCDBR/MdARL2 (Hn2) lines and the 'Royal Gala' control. Expression was determined by qRT-PCR using primers (**Table S5**) specific for *MdHCDBR* (left) and *MdARL2* (right). Expression in the control (black bars) is set at 100%. Data are means +/- SE, n=3 biological replicates. The lines shown as red bars were selected for further characterization by qPCR and HPLC. Statistical analysis was performed in GraphPad Prism: one-way ANOVA using Dunnett's Multiple Comparison Test *vs* control, P<0.001 = *. (B) Relative concentration of phlorizin in the selected Hn2 transgenic leaves and the 'Royal Gala' control measured by HPLC. Concentration in the control (black bar) is set at 100%. Data are means +/- SE, n=3 biological replicates. Statistical analysis was performed in GraphPad Prism: one-way ANOVA using Dunnett's Multiple Comparison Test *vs* control. No significant difference in phlorizin content was observed. (C) Relative expression of *MdARL1* and *MdARL3* in the leaves of four selected transgenic Hn2 lines and the 'Royal Gala' control. Expression was determined by qRT-PCR using primers (**Table S5**) specific for *MdARL1* (left) and *MdARL3* (right). Expression in the control (black bars) is set at 100%. Data are means +/- SE. Statistical analysis

was performed in GraphPad Prism: one-way ANOVA using Dunnett's Multiple Comparison Test *vs* control, P<0.001 = *.

(A) Alignment of NCR amino acid sequences

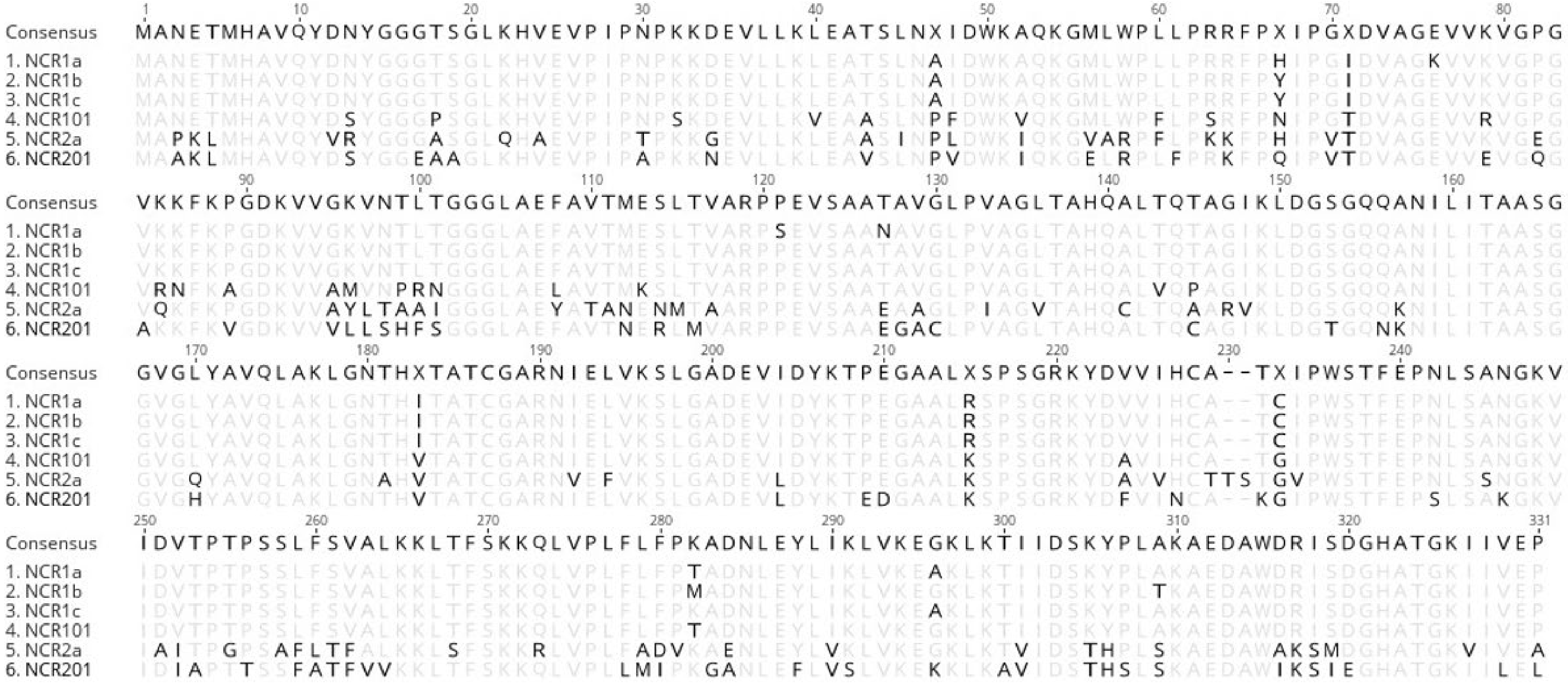

(B) Phylogram of NCR gene models from Maleae and Gillenieae

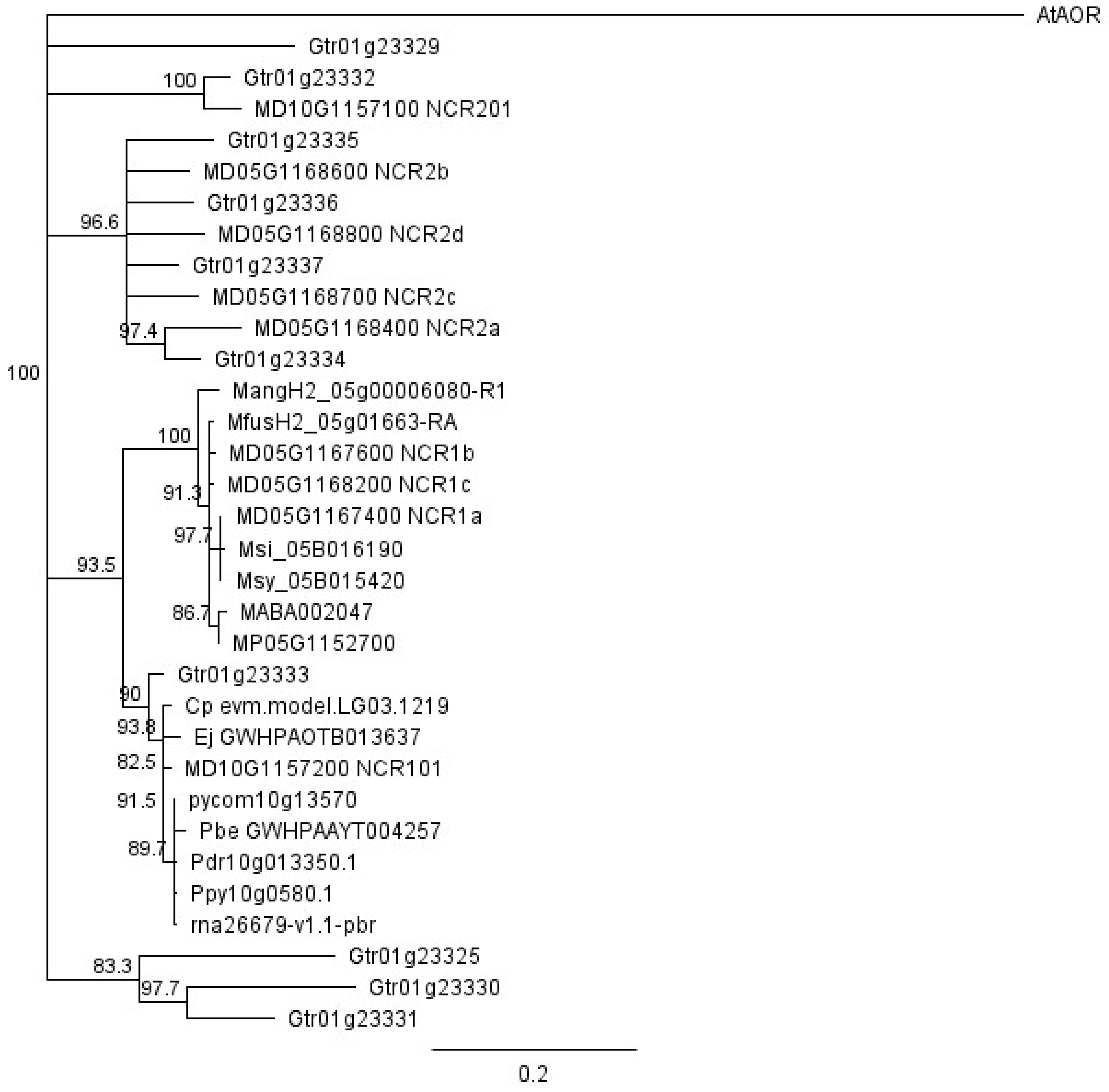

(C) Representative UPLC traces of *MdNCR1* inoculations into *N. benthamiana*

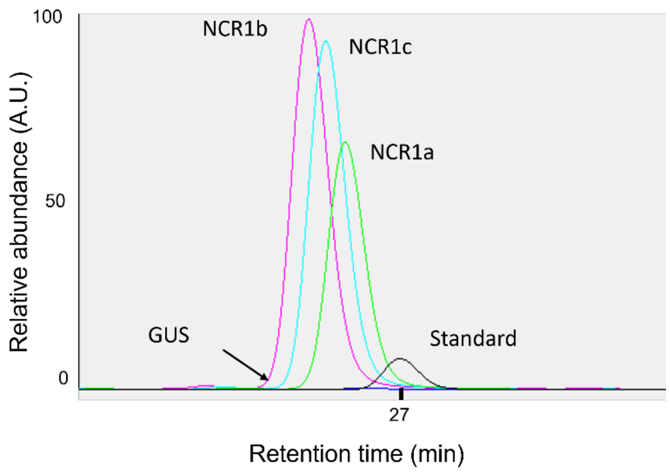

Supplementary Figure S3. NCR alignment, phylogeny and additional UPLC trace.
(A) Amino acid sequences from six NCR gene models were aligned using Clustal Omega in Geneious Prime (Version 2022.0.1). Differences to the consensus are highlighted. (B) Phylogram of NCR gene models from Maleae and Gillenieae. The nearest homolog to *MdNCR1b* was obtained using BLASTP in six sequenced *Malus* spp. genomes, five sequenced *Pyrus* spp. genomes, Chinese hawthorn (*Craetagus pinnatifida*) and loquat (*Eriobotrya japonica*) at the Genome Database for Rosaceae (Jung et al., 2019; Nucleic Acids Res **47:** D1137-D1145). NCR homologs in the *Gillenia trifoliata* genome were obtained by BLASTP with a cutoff of $<e^{-90}$. Amino acid alignments were generated with the MUSCLE alignment tool in Geneious Prime (Version 2022.0.1). Trees were inferred using the Maximum Likelihood method based on the JTT matrix-based model (Jones et al., 1992, Comput Appl Biosci **8:** 275-282). AtAOR (Q9ZUC1.2) was used as the outgroup. Percentage bootstrap values >80% (1000 replicates) are shown. Branch lengths measure the number of substitutions per site relative to the scale bar. *M.* × *domestica* (MD), *M. angustifolia* (Mang), *M. fusca* (Mfus), *M. prunifolia* (MP), *M. sieversii* (Msi), *M. baccata* (MAB), *M. sylvestris* (Msy), *P. ussuriensis* PDR, *P. pyrifolia* (Ppy), *P. communis* (pycom), *P.* × *bretschneideri* (pbr), *P. betulifolia* (pbe), *C. pinnatifida* (cp), *E. japonica* (Ej), and *G. trifoliata* (Gtr). (C) Representative UPLC traces of *MdNCR1* inoculations into *N. benthamiana*. All inoculations were performed in combination with pHEX2_PGT1 and pHEX2_MYB10. *MdNCR1a* (green, 5× dilution), *MdNCR1b* (purple, 5× dilution), *MdNCR1c* (light blue, 5× dilution), phlorizin standard (black) and *GUS* as the negative control (dark blue). Traces are offset to the phlorizin standard running at 27 min. for clarity. Relative abundance in absorbance units (A.U.) with MdNCR1b set as the maximum.

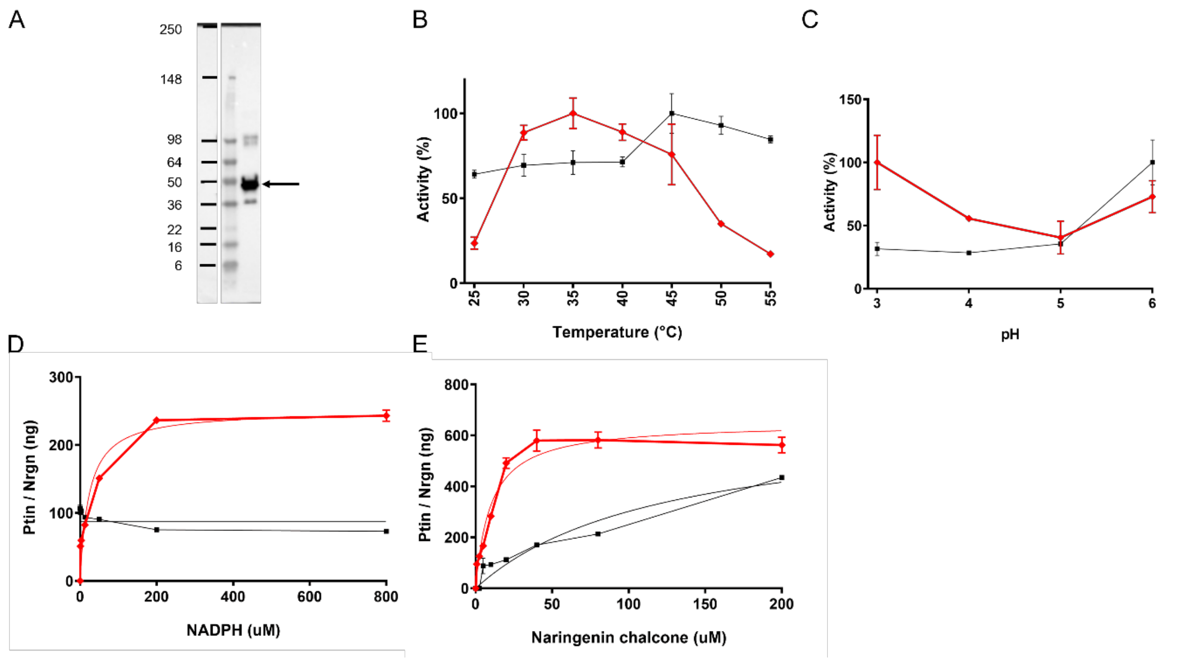

Supplementary Figure S4. Purification of recombinant MdNCR1 and optimizing NCR reaction conditions.

(A) Immunodetection of MdNCR1b using a His_6 monoclonal antibody (PharMingen, www.bdbiosciences.com). Recombinant MdNCR1b protein was produced in *Escherichia coli*, purified by Ni^{2+} affinity chromatography and gel filtration and proteins separated by SDS-PAGE. Ladder = SeeBlue Plus ladder (Thermo Scientific). Arrow indicates the position of the recombinant MdNCR1b band. The optimal temperature (B) and pH (C) conditions for measuring NCR enzyme activity were determined using recombinant MdNCR1b enzyme with naringenin chalcone (100 µM) and NADPH (1.25 mM) using the NCR activity assay conditions described in Methods. The plots in (D) and (E) were used to determine K_m and V_{max} for naringenin chalcone and NADPH respectively. Data are mean ± SE, n=3. Red line is NCR conversion of naringenin chalcone to phloretin (Ptin), black line is spontaneous isomerization of naringenin chalcone to naringenin (Nrgn).

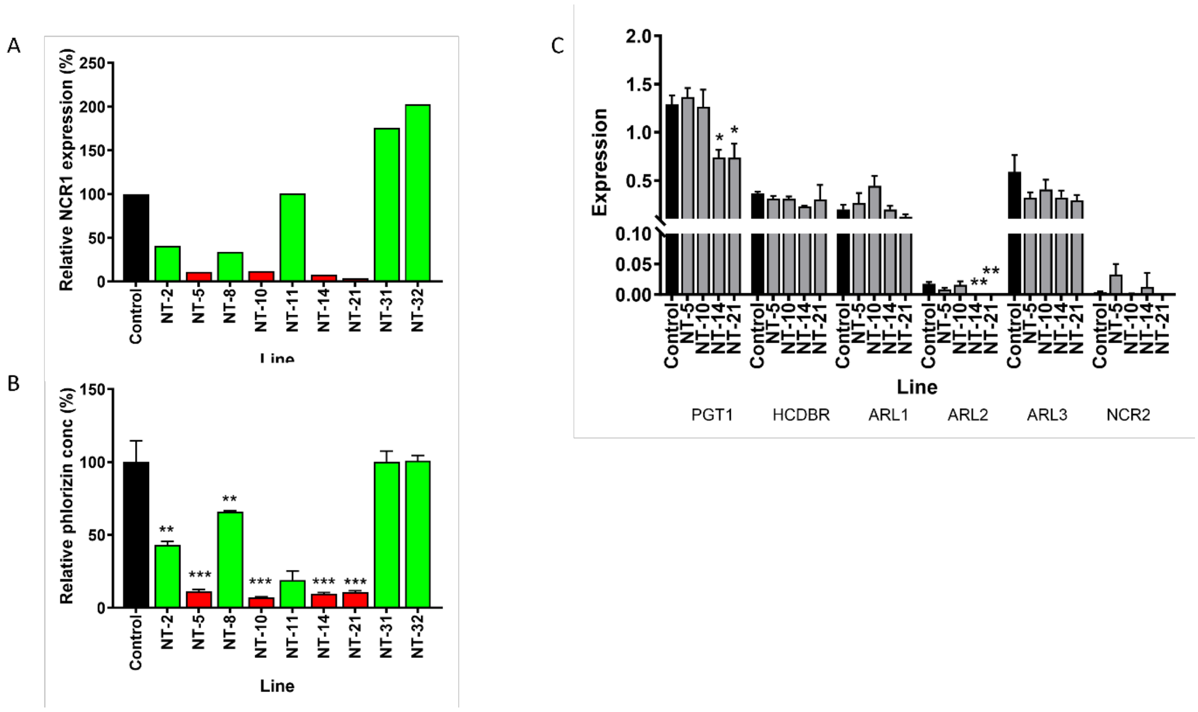

Supplementary Figure S5. Screening of transgenic NCR lines by qPCR and HPLC. (A) Relative expression of *MdNCR1* in leaves of NCR transgenic lines (NT) and the 'Royal Gala' control. Expression was determined by qRT-PCR using NCR1-specific primers (**Table S5**). Expression of the control (black bar) is set at 100%. As a rapid screen only one biological replicate was used, therefore no statistical analysis was performed. (B) Relative concentration of phlorizin in NT transgenic leaves and the 'Royal Gala' control' measured by HPLC. Expression of the control (black bar) is set at 100%. Data are means +/- SE, n=3 biological replicates. Statistical analysis was performed in GraphPad Prism: one-way ANOVA using Dunnett's Multiple Comparison Test *vs* control, P<0.1 = *, P<0.01 = **, P<0.001 = ***. The lines shown as red bars were selected for further characterization. (C) Expression of DHC-related genes in the leaves of four transgenic NCR lines and the 'Royal Gala' control. *MdPGT1*; *MdHCDBR*; *MdARL1–3* and *MdNCR2*. Data are means +/- SE, n=3 biological replicates. Statistical analysis was performed in GraphPad Prism: one-way ANOVA using Dunnett's Multiple Comparison Test *vs* control, P<0.1 = *, P<0.01 = **, P<0.001 = ***.

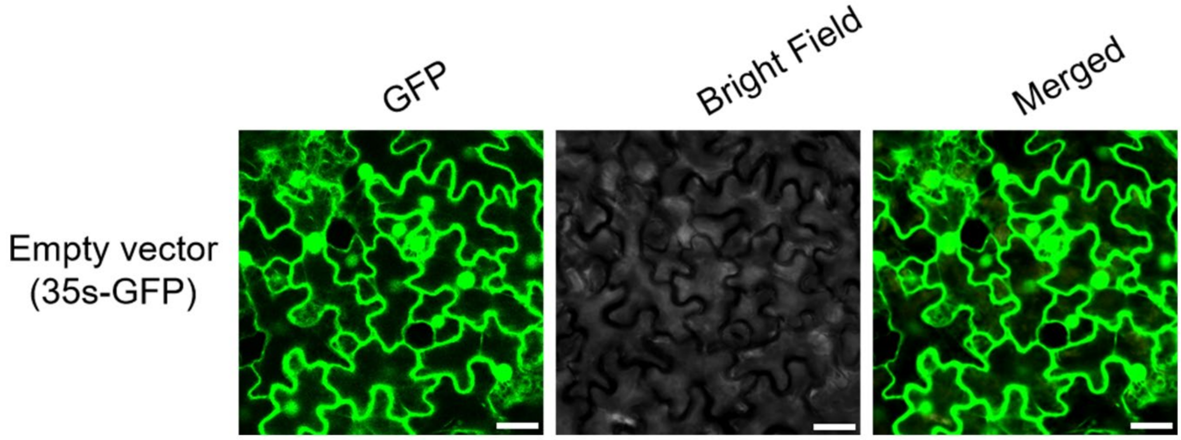

Supplementary Figure S6. Subcellular localization of 35S-GFP in *N. benthamiana*. The control construct 35S-GFP was transiently expressed in *N. benthamiana* leaves and analyzed with a confocal laser-scanning microscope. GFP, GFP fluorescence; Brightfield, light microscopy images; Merged, GFP and Brightfield images merged. Fluorescence is observed throughout the cell, including the cytoplasm and membranes. Scale bars, 25 µm.

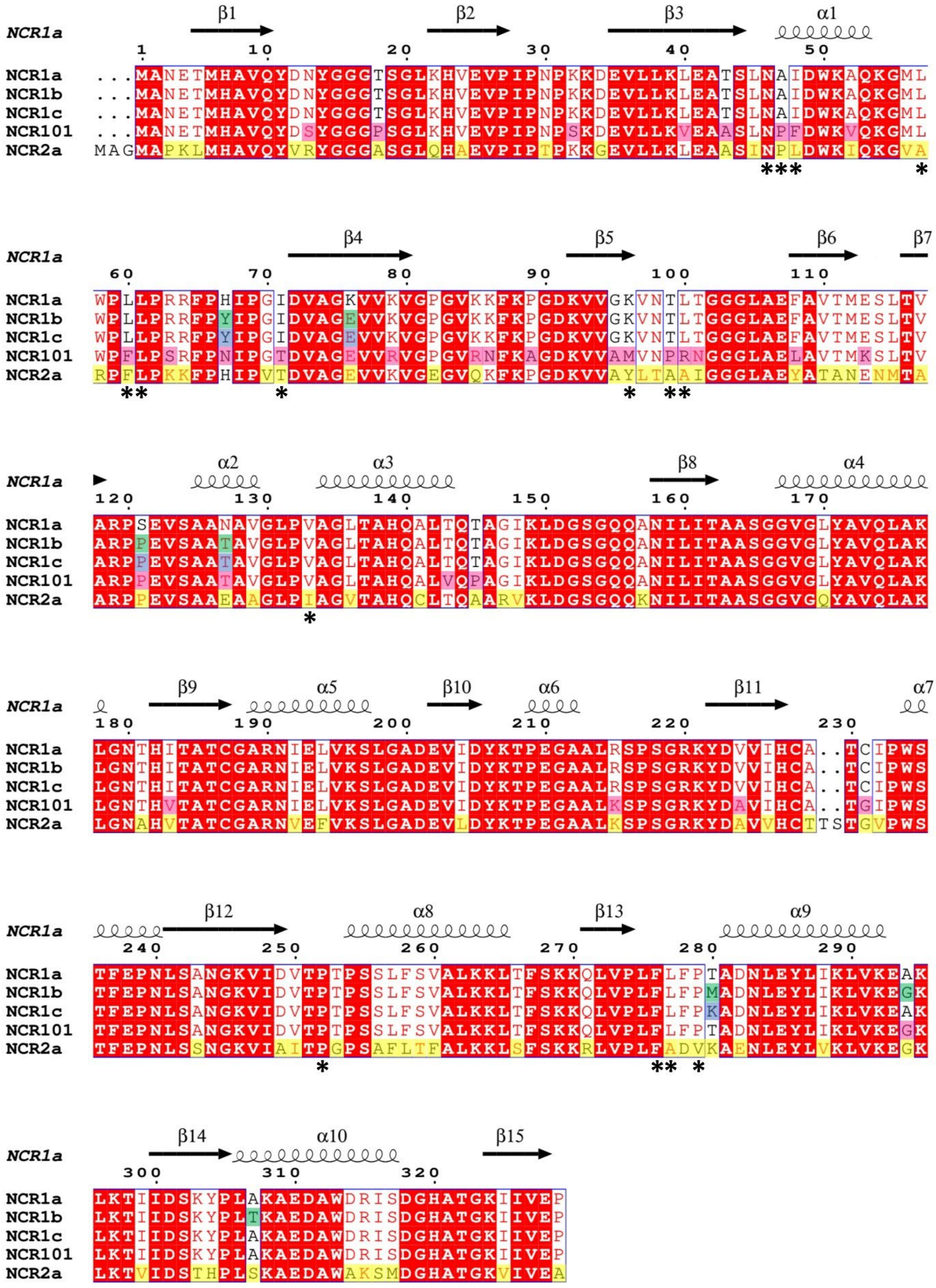
NCR1a
NCR1b
NCR1c
NCR101
NCR2a

Supplementary Figure S7. Sequence alignment between MdNCR1a–c, MdNCR101 and MdNCR2a.

The topology of the MdNCR1a model is shown above the alignment. Strictly conserved residues are highlighted in red. Variations between MdNCR1a and MdNCR1b, MdNCR1c, MdNCR101, and MdNCR2 are highlighted in green, blue, pink, and yellow, respectively. Residues lining the substrate binding pocket are indicated by a star.

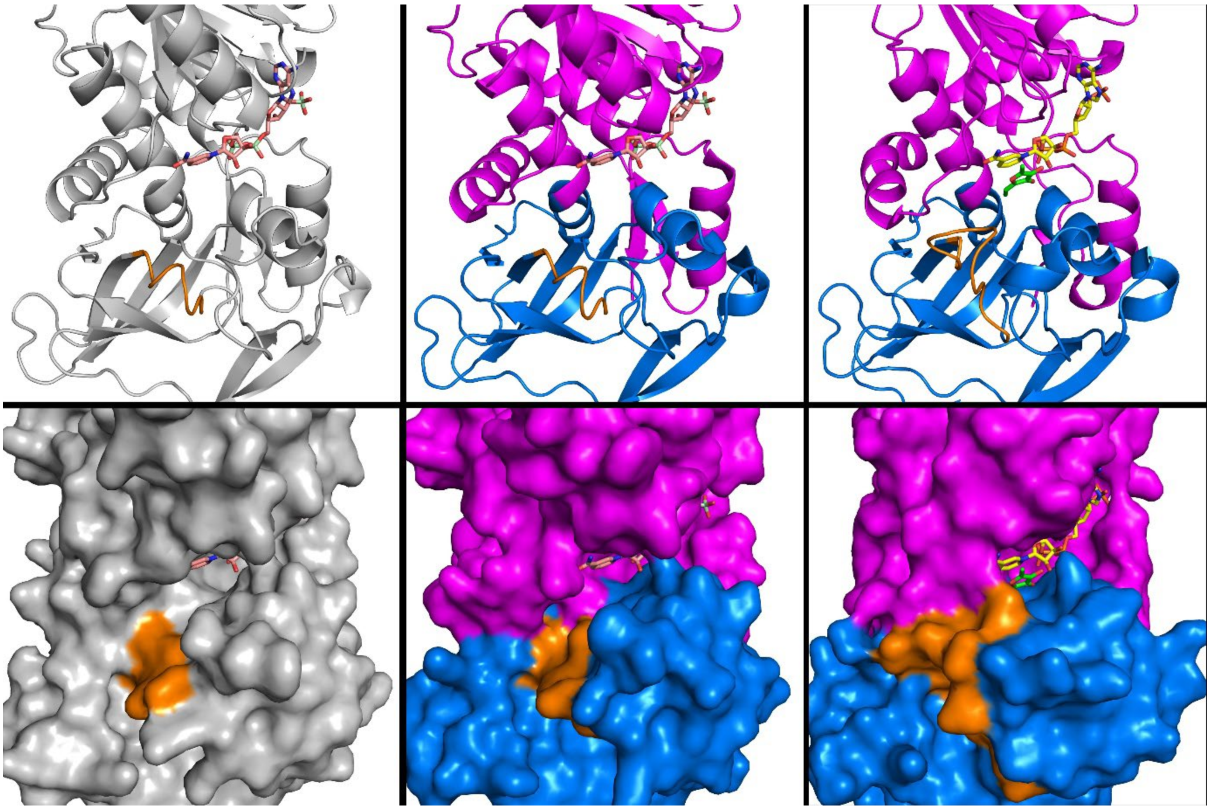

Supplementary Figure S8. Structural comparisons between the MdNCR1a–c, AtceQORH and FaEO.

Structural comparisons between the MdNCR1a–c model (left) and the crystal structures of the *Arabidopsis thaliana* chloroplast envelope quinone oxidoreductase AtceQORH (PDB entry 5A4D, middle) and of the strawberry oxidoreductase FaEO (PDB code 4IDE, right). Top panels show the proteins in ribbon mode, bottom panels show their corresponding surface representations. The catalytic and substrate binding domains of AtceQORH and FaEO are shown in magenta and blue, respectively. The NADP moiety of AtceQORH is shown in stick mode, with carbon atoms in pink and phosphate atoms in pale green, respectively. The NADP and (2E)-ethylidene-4-hydroxy-5-methyl-3(2H)-furanone (EDHMF) moieties of FaEO are shown in stick mode, with carbon atoms in yellow and phosphate atoms in orange for NADP, and carbon atoms in green for EDHMF. Nitrogen and oxygen atoms are in blue and red, respectively. The loops shaping the size of the substrate binding pockets (residues 96–104 in MdNCR1a–c and AtceQORH, residues 103–114 in FaEO) are highlighted in orange.

Supplementary Method S1. NCR product analysis by GC-MS analysis

Volatile products from the NCR enzyme reactions were extracted using 0.2 mL of pentane:diethyl ether (1:1). Cyclohexanone (2 µL of a 1 $\mu g \cdot \mu L^{-1}$ stock) was added as the internal standard. Extractions were performed at room temperature for 2 h with gentle shaking every 20–30 min. Sample vials were stored at −20°C overnight and the solvent phase was carefully isolated and 1 µL injected into the GC TOF-MS for volatile analysis.

The GC column used was a DB-HeavyWax column (30 cm x 0.25 mm x 0.25 µm, Agilent). GC flow was operated in 20 to 1 split mode, with a flow rate at 1.0 $mL \cdot min^{-1}$. The GC temperature was held at 40°C for 1 min followed by temperature ramps to 150°C at 10°C·min^{-1} and then to 240°C at 15°C·min^{-1}. The final temperature was held for 12 min, giving a total analysis time of 30 min. Volatile compounds were detected by BT-TOF-MS. The transfer line temperature was set at 240°C with 70 V of electron energy. Mass spectra from 35 to 400 atomic mass units were collected with a data acquisition rate of 25 $spectra \cdot s^{-1}$. The total ion and selected ion chromatograms were processed using LECO Chroma TOF software (Version 5.50 Leco Australia) for relative quantitation and identification of detected volatile metabolites.

Compound identification was by ChromaTOF software (Version 5.50, Leco, Australia) using in-house reference compounds, the National Institute of Standards and Technology (NIST) mass-spectral database (Version 2.4), and retention indices calculated relative to a series of straight-chain hydrocarbon standards (C7–C30). Peaks were selected and integrated automatically using diagnostic ions for each compound. Relative concentrations of volatiles ($ng \cdot g^{-1}$) were calculated in comparison with the amount of cyclohexanone added to each sample.

Supplementary Table S1. Expression and amino acid identities of apple MdHCDBR and MdARL genes.
(A) Expression of MdHCDBR and MdARL genes in apple. Leaf transcriptome data were downloaded from the Apple Multidimensional Omics Database (bioinformatics.cau.edu.cn/AppleMDO/). DPI = days post infection. The four MdARL genes expressed at an average RPKM >10 in leaf are indicated in green. (B) Amino acid sequences of apple MdHCDBR and four MdARL gene models from the 'Golden Delicious' apple genome were aligned using Clustal Omega in Geneious (Version 10.0.3). Percentage amino acid identity between the genes is shown.

(A)

Gene name	Gene model	SRR767660-leaf uninfected	SRR767669-leaf 2DPI	SRR767670-leaf 4DPI	SRR767671-leaf 6DPI	SRR767672-leaf 8DPI	SRR767673-leaf 10DPI	SRR767674-leaf 12DPI	SRR768127-leaf 14DPI	SRR1089478-leaf	SRR1089477-leaf	SRR3744769-leaf	SRR3744770-leaf	SRR3744771-leaf	SRR5405145-leaf	SRR6320561-leaf	SRR6308182-young_leaves	Leaf average
HCDBR	MD15G1145700	123	75	84	168	80	121	134	109	82	74	97	93	93	57	92	35	95
ARL1	MD02G1002400	7	0	3	16	3	9	25	16	10	8	46	42	43	10	22	7	17
ARL3	MD02G1001800	93	40	46	97	119	65	49	41	23	26	46	43	42	77	81	6	56
ARL2	MD12G1260900	18	29	20	37	18	25	17	12	13	12	7	7	7	9	15	49	18
ARL4	MD01G1046700	12	0	6	11	0	5	6	4	2	2	3	4	4	6	10	0	5
ARL7	MD00G1045700	0	0	0	0	0	0	3	0	0	0	0	0	0	0	0	0	0

(B)

Gene name	ARL1	ARL3	ARL2	ARL4	ARL7
HCDBR	93.5%	93.5%	64.0%	71.3%	65.3%
ARL1		94.4%	64.1%	71.3%	66.6%
ARL3			63.5%	70.1%	65.8%
ARL2				62.1%	59.3%
ARL4					67.2%

Supplementary Table S2. Identification of NCR gene candidates.

To identify NCR gene candidates, apple (MD) gene models were obtained from Genome Database for Rosaceae (Jung et al., 2019; Nucleic Acids Res **47:** D1137-D1145) and the annotations screened for the words reductase or dehydrogenase. Transcriptome data for each gene model from a range of apple tissues were downloaded from the Apple Multidimensional Omics Database (bioinformatics.cau.edu.cn/AppleMDO/). Screen 1: Average RPKM values were obtained for all database samples annotated as coming from leaves (yellow) and from fruit older than 35 DAP (days after pollination) or 5 WAFB (weeks after full bloom) (yellow). Gene models shown exhibit an average RPKM >10 in leaves and leaf/fruit expression >4. Screen 2: Gene models shown exhibit an average RPKM >10 in leaves, leaf/fruit expression >4 and an average RPKM <10 in fruit. Screen 3: Gene models shown exhibit an average RPKM >10 in leaves, leaf/fruit expression >4, average RPKM <10 in fruit and average RPKM >10 in stems and young fruit (orange). The three NCR gene models identified for further characterization are highlighted in red.

Excel table is available as a separate attachment.

Supplementary Table S3. Genomic location, open reading frame size and gene annotation of each NCR gene model. Apple gene models, start and end co-ordinates, open reading frame size (ORF, in amino acids) and annotation were obtained from Genome Database for Rosaceae (Jung et al., 2019; Nucleic Acids Res **47:** D1137-D1145). Full-length *MdNCR1/101* gene models are highlighted in yellow and full-length *MdNCR2/201* gene models in green.

Chr 05	Name	Start	End	ORF	Annotation
MD05G1166800		29700093	29701066	224	putative quinone-oxidoreductase homolog, chloroplastic [Pyrus x bretschneideri];hypothetical protein PRUPE_ppa019985mg [Prunus persica];hypothetical protein CICLE_v10021072mg [Citrus clementina]
MD05G1167000		29703900	29704886	228	putative quinone-oxidoreductase homolog, chloroplastic [Pyrus x bretschneideri, Prunus mume];hypothetical protein PRUPE_ppa016022mg [Prunus persica];hypothetical protein PRUPE_ppa008358mg [Prunus persica]
MD05G1167200		29715730	29716365	211	putative quinone-oxidoreductase homolog, chloroplastic [Prunus mume, Malus domestica];hypothetical protein PRUPE_ppa025348mg [Prunus persica];hypothetical protein PRUPE_ppa018887mg [Prunus persica]
MD05G1167300		29717650	29718779	135	putative quinone-oxidoreductase homolog, chloroplastic [Pyrus x bretschneideri, Prunus mume, Malus domestica];hypothetical protein PRUPE_ppa015290mg [Prunus persica];putative quinone-oxidoreductase homolog, chloroplastic-like [Fragaria vesca subsp. vesca]
MD05G1167400	NCR1a	29748770	29751777	329	putative quinone-oxidoreductase homolog, chloroplastic [Pyrus x bretschneideri, Prunus mume, Malus domestica];hypothetical protein PRUPE_ppa008491mg [Prunus persica];hypothetical protein PRUPE_ppa008401mg [Prunus persica]
MD05G1167600	NCR1b	29783033	29785905	329	putative quinone-oxidoreductase homolog, chloroplastic [Pyrus x bretschneideri, Prunus mume, Malus domestica];hypothetical protein PRUPE_ppa008491mg [Prunus persica];putative quinone-oxidoreductase homolog, chloroplastic-like [Fragaria vesca subsp. vesca]
MD05G1167700		29789662	29791685	71	quinone-oxidoreductase homolog, chloroplastic-like [Malus domestica];putative quinone-oxidoreductase homolog, chloroplastic [Pyrus x bretschneideri, Malus domestica]
MD05G1168000		29832929	29833412	103	putative quinone-oxidoreductase homolog, chloroplastic [Pyrus x bretschneideri, Prunus mume, Malus domestica];hypothetical protein PRUPE_ppa015290mg [Prunus persica];putative quinone-oxidoreductase homolog, chloroplastic-like [Fragaria vesca subsp. vesca]
MD05G1168100		29833721	29833924	67	putative quinone-oxidoreductase homolog, chloroplastic [Malus domestica, Eucalyptus grandis, Camelina sativa];Putative quinone-oxidoreductase-like protein [Morus notabilis];hypothetical protein CICLE_v10021072mg [Citrus clementina]

MD05G1168200	NCR1c	29835084	29838274	329	putative quinone-oxidoreductase homolog, chloroplastic [Pyrus x bretschneideri, Prunus mume, Malus domestica];hypothetical protein PRUPE_ppa008491mg [Prunus persica];putative quinone-oxidoreductase homolog, chloroplastic-like [Fragaria vesca subsp. vesca]
MD05G1168400	NCR2a	29855406	29857998	334	putative quinone-oxidoreductase homolog, chloroplastic [Pyrus x bretschneideri, Malus domestica];putative quinone-oxidoreductase homolog, chloroplastic isoform X1 [Pyrus x bretschneideri];putative quinone-oxidoreductase homolog, chloroplastic isoform X2 [Pyrus x bretschneideri]
MD05G1168500		29858409	29859628	54	putative quinone-oxidoreductase homolog, chloroplastic, partial [Prunus mume];hypothetical protein PRUPE_ppa008358mg [Prunus persica];putative quinone-oxidoreductase homolog, chloroplastic [Camelina sativa]
MD05G1168600	NCR2b	29860406	29862383	331	putative quinone-oxidoreductase homolog, chloroplastic [Pyrus x bretschneideri, Prunus mume, Malus domestica];hypothetical protein PRUPE_ppa008401mg [Prunus persica];putative quinone-oxidoreductase homolog, chloroplastic-like [Fragaria vesca subsp. vesca]
MD05G1168700	NCR2c	29863248	29866278	329	putative quinone-oxidoreductase homolog, chloroplastic [Pyrus x bretschneideri, Prunus mume, Malus domestica];hypothetical protein PRUPE_ppa008401mg [Prunus persica];putative quinone-oxidoreductase homolog, chloroplastic isoform X1 [Pyrus x bretschneideri]
MD05G1168800	NCR2d	29867598	29870249	330	putative quinone-oxidoreductase homolog, chloroplastic [Pyrus x bretschneideri, Prunus mume, Malus domestica];hypothetical protein PRUPE_ppa008401mg [Prunus persica];putative quinone-oxidoreductase homolog, chloroplastic-like [Fragaria vesca subsp. vesca]
**Chr 10**					
MD10G1157000		24569711	24571385	293	putative quinone-oxidoreductase homolog, chloroplastic [Pyrus x bretschneideri];hypothetical protein PRUPE_ppa018887mg [Prunus persica];hypothetical protein PRUPE_ppa025348mg [Prunus persica]
MD10G1157100	NCR201	24577598	24579632	329	putative quinone-oxidoreductase homolog, chloroplastic [Pyrus x bretschneideri, Prunus mume, Malus domestica];hypothetical protein PRUPE_ppa015290mg [Prunus persica];putative quinone-oxidoreductase homolog, chloroplastic-like [Fragaria vesca subsp. vesca]
MD10G1157200	NCR101	24579635	24582423	329	putative quinone-oxidoreductase homolog, chloroplastic [Pyrus x bretschneideri, Prunus mume, Malus domestica];hypothetical protein PRUPE_ppa008491mg [Prunus persica];putative quinone-oxidoreductase homolog, chloroplastic-like [Fragaria vesca subsp. vesca]

Supplementary Table S4. Amino acid identities and targeting predictions for apple NCR genes.

(A) Amino acid sequences of the nine complete NCR gene models from the ‘Golden Delicious’ apple genome were aligned using Clustal Omega in Geneious (Version 10.0.3). Percentage amino acid identity between the apple NCR genes is shown. (B) TargetP 2.0 predictions from https://services.healthtech.dtu.dk/services/TargetP-2.0/. (C) Alignment of the 41 amino acid cryptic chloroplast targeting region in AtceQORH Curien et al., 2016; Phytochem **122:** 45-55) to AtAOR and five apple NCRs. Residues in bold are conserved.

(A)

	NCR1a	NCR1b	NCR1c	NCR201	NCR2a	NCR2b	NCR2c	NCR2d
NCR101	85.7%	86.3%	86.6%	75.1%	74.6%	79.0%	78.1%	77.8%
NCR1a		98.2%	98.2%	73.3%	72.5%	77.2%	76.9%	76.3%
NCR1b			99.1%	73.9%	72.8%	78.1%	77.2%	76.6%
NCR1c				74.2%	72.8%	78.1%	77.2%	76.6%
NCR201					72.8%	78.1%	78.7%	77.5%
NCR2a						84.1%	82.2%	84.0%
NCR2b							87.8%	88.8%
NCR2c								88.1%
NCR2d								

(B)

	Other	Signal peptide	Mitochondrial transfer peptide	Chloroplast transfer peptide	Thylakoid luminal transfer peptide
NCR1a	0.9998	0.0002	0	0	0
NCR1b	0.9998	0.0002	0	0	0
NCR1c	0.9998	0.0002	0	0	0
NCR101	0.9995	0.0004	0	0	0
NCR2a	0.8502	0.0027	0.1409	0.0062	0
AtceQORH	0.9516	0.0025	0.035	0.011	0
AtAOR	0.0021	0	0.0043	0.9283	0.0653
AtAER	0.9971	0.0026	0.0003	0	0
FaEO	0.9996	0.0003	0	0	0
CsChlAOR	0.0001	0	0	0.992	0.0079
CsAOR	0.9924	0.0008	0.0001	0.0066	0

(C)

Protein	Cryptic chloroplast targeting region	Total	% identity
AtceQORH	FLPRKFPCIPATDVAGEVVEVGSGVKNFKAGDKVVAVLSHL		
AtAOR	TDS-PL**P**TV**P**GY**DVAG**V**VV**K**VGS**A**VK**DL**K**E**GD**E**V**Y**A**NV**S**EK	19/41	46
NCR1a	L**LPR**R**FP**H**IP**GI**DVAG**K**VV**K**VG**P**GVK**K**FK**P**GDKVV**GKVNT**L**	26/41	63
NCR1b	L**LPR**R**FP**Y**IP**GI**DVAGEVV**K**VG**P**GVK**K**FK**P**GDKVV**GKVNT**L**	27/41	66
NCR101	**FLP**SR**FP**N**IP**G**TDVAGEVV**R**VG**P**GV**R**NFKAGDKVVA**MVNPR	29/41	71
NCR2a	**FLPKKFP**H**IP**V**TDVAGEVV**K**VG**E**GVQKFK**P**GDKVVA**Y**L**TAA	32/41	78

Supplementary Table S5. Oligonucleotide primers used for RT-qPCR and cloning.

Restriction sites and attB1 and attB2 sequences in primers used for cloning are underlined. nt = not tested.

RT-qPCR

Target	Forward primer (5′ → 3′)	Reverse primer (5′ → 3′)	Primer efficiency	Product size (bp)
NCR1	AGCTGCTTCAGGTGGTGTAG	GACCAGTTCAATGTTGCGGG	1.88	100
NCR2	AATGTCTCACGCAAGCTGCT	ACTGCATACTGACCCACACC	1.91	100
NCR101	AGTTCCGATTCCAAATCCGAGC	CCTCCATTACGAGGGTTAACCATTG	1.94	234
ARL2	CCGAGGGATCCGCAATGTAT	TTCCAAGAAGCGTGGGTACA	1.88	100
HCDBR	AAATGGTTCTGCCGGCGATA	GCATAGAGCCCTATCAGAGCG	1.78	100
ARL1	ACTTTCCTGAAGGCATCGACA	CAAACTGCAATCCGGCCATG	1.98	100
ARL3	ATGTCGGGGGAAAGATGCTT	CGCTGGTCAAGGGTGTACTG	1.86	100
4CL	CATAAACAGTGTCCCCAAGTCAGCAT	AGTGTTCCTACAAGCCTTCCCGATAA	1.82	142
CHI2	CGTTTCCACCGTCCGCCAAA	TCTTACCCTTCCACTTAACGGCGA	1.45	168
CHS2	CAGCGTTGATTTATCTATCTGCTTCTGC	TGCACCAAGTTAACCCCATGACG	1.90	133
PGT1	GAAGGGTGTGTTGCCAGAAGGGT	GTCACGAACCCACCAACCGACT	1.92	117
UFGT	CCACCGCCCTTCCAAACACTCT	CACCCTTATGTTACGCGGCATGT	1.98	107
FLS	CAGGACCACTGTGAACAAGGA	GTTCACAAACCGTGGATGAGG	1.94	100
F3H	TGGAAGCTTGTGAGGACTGGGGT	CTCCTCCGATGGCAAATCAAAGA	1.69	116
MdEF1α	CCAAGATTGACAGGAGGTCTGGAAA	ATGCAGGTATGGTGAAGATGCTTCC	1.85	88

Cloning

Target	Forward primer (5′ → 3′)	Reverse primer (5′ → 3′)	Purpose
NCR1	AAAAAAGCAGGCTCCATGGCCAATGAGACCATGCA	AGAAAGCTGGGTCTAAGGCTCCACAATTATCT	cDNA into pDONR/pHEX2/pET300
NCR2	AAAAAAGCAGGCTCCATGGCGCCCAAGCTTATGCA	AGAAAGCTGGGTTTAAGCCTCAACGATGACCT	cDNA into pDONR/pHEX2/pET300
HCDBR	AAAAAAGCAGGCTCCATGGCGGCAAGTACAGAGGG	AGAAAGCTGGGTTCATTCACGGGAAACCACAA	pHEX2 into pET300
ARL2	AAAAAAGCAGGCTCCATGGCTGAAAAAGTACAAGT	AGAAAGCTGGGTTCATTCGTGGGCTACACGAA	cDNA into pDONR
NCR1a,b & NCR101	CGAGCTCGGTACCCGGGGATCCATGGCCAATGAGACCATGCATGC	CCTTGCTCACCATGGTGTCGACAGGCTCCACAATTATCTTTCCGG	pDONR into pCAMBIA2300-GFP
NCR2	CGAGCTCGGTACCCGGGGATCCATGGCAGGCATGGCGCCC	CCTTGCTCACCATGGTGTCGACAGCCTCAACGATGACCTTCCCG	pDONR into pCAMBIA2300-GFP
