# Supplementary material for: Naringenin chalcone carbon double-bond reductases mediate dihydrochalcone biosynthesis in apple leaves
Source: Plant Physiol. 2024 Sep 29;196(4):2768–83. doi: 10.1093/plphys/kiae515 (PMC11638483; doi:10.1093/plphys/kiae515)
Supplement: kiae515_Supplementary_Data [file pp.00532.2024-s06.pdf › pp.00532.2024-s06.pdf]

Plant Physiology®

Naringenin chalcone carbon double-bond reductases are required for dihydrochalcone biosynthesis in apple leaves

Yar-Khing Yauk, Andrew P. Dare, Janine Cooney, Yule Wang, Cyril Hamiaux, Tony McGhie, Mindy Wang, Pengmin Li, and Ross Atkinson

Review Timeline:

Submission Date:	09-Apr-2024
Editorial Decision:	14-May-2024
Revision Received:	25-Jul-2024
Editorial Decision:	12-Aug-2024
Revision Received:	03-Sep-2024
Accepted:	04-Sep-2024

14-May-2024

Dr. Ross G. Atkinson
New Zealand Institute for Plant and Food Research Ltd
Fruit Quality
Auckland, n/a 1141
New Zealand

RE: A naringenin chalcone carbon double-bond reductase is the missing enzyme in dihydrochalcone biosynthesis in apple leaves

Dear Dr. Atkinson:

We have received reviews of your manuscript, which you may view at the end of this letter. On the basis of the advice received and my own evaluation, I believe that, pending revision, this article may ultimately be acceptable for publication in Plant Physiology.

As you can see, the reviewers question whether the RNAi downregulation is specific to NCR for the observed reduction of phlorizin level and why not do the transient overexpression in apple. Perhaps a better image of subcellular localization is needed.

When you are ready to submit your revision, click the below link and find the red arrow under your Author Tasks heading for this revision submission.

If you want to visit this version of the manuscript, you may click the link below and then click on the folder that reads "Post Decision Manuscripts".

Link Not Available

When uploading the revised version of this article, please be sure to include as complete an answer as possible to the referees' criticisms during the first round of reviews in the "response to reviewers" section.

Plant Physiology considers a revision for up to 60 days after a manuscript has been provisionally accepted; limited extension of this timeframe is at the discretion of the Monitoring Editor. A reminder notice will be sent to you 2 weeks before the deadline, at which time you may choose to contact the editorial office to request an extension; if so, please provide a justification for your request. After the expiration of this deadline, the article would need to be submitted as a resubmission and would be handled as such.

Thank you very much for giving us an opportunity to review this work. I look forward to receiving the next version.

Sincerely,

Editor, Plant Physiology

Want to add this revision deadline to your calendar? Click below!

-- Reviewer comments:
Reviewer #1 (Comments for the Author):

In this research, the authors discovered the synthesis of Dihydrochalcones (DHCs) in apples via the phenylpropanoid pathway, highlighting MdHCDBR and MdNCR1a/b as pivotal components. While reducing MdHCDBR did not impact DHC levels, overexpressing MdNCR1a/b facilitated DHC production. Particularly, MdNCR1b efficiently transformed naringenin chalcone into phloretin. Suppressing MdNCR1 in apples decreased DHC levels, favoring the production of flavonol glycosides. Additionally, the likely vacuolar membrane localization of MdNCR1 sheds light on DHC regulation, offering promising implications for biotechnological advancements. I think the findings will be of interest to readers of Plant Physiology, especially those who are

interested in phenylpropanoid pathway. I also think that the discoveries will provide valuable information into the field, however, at this time, the manuscript submitted seems to be premature and need to be improved.

1. My major comment is that the discussion section should be improved, it seems more a conclusion than a discussion section, and the amount of data and findings here described deserve further discussion.
2. Why should MdHCDBR, MdARL2, and their homologous genes all be knocked down through RNAi interference, and do they exhibit differential expression levels and potential functional redundancy among them? Please comment.
3. How many NCR genes are present in apple, do they share similar functions, and are there any intriguing evolutionary events related to NCR genes in the Rosaceae family? Please elaborate further on this topic.

Reviewer #2 (Comments for the Author):

The manuscript by Yauk et al. reported a key reaction step catalyzed by a novel enzyme naringenin chalcone reductase during the biosynthesis of dihydrochalcones. Using a combination of transient expression, in vitro enzyme assays, RNAi transgenic analyses, the authors established that phloretin is synthesized primarily through direct reduction of naringenin chalcone in apple leaves. Overall, the experiments were designed logically and data presented were quite conclusive. Nonetheless, there are a few issues that require clarification before the manuscript is acceptable.

Major issues:
(1) Downregulation of NCR genes in transgenic apple leaves resulted in decreased phlorizin production but increased levels of quercetin glycosides. Surprisingly, 4CL and CHS gene expressions were also down-regulated. As 4CL and CHS produce precursors for both flavonoids and dihydrochalcones, both types of metabolites should be reduced in abundance. In fact, the CHS downregulated line A9 showed 50% reduction in NCR1 expression with 90% reduction in both DHC and flavonoids (Line 295-297). The authors should address this issue in more detail. For example, was the RNAi downregulation in their transgenic apple specific to NCR genes only? Was the reduced phlorizin level directly caused by NCR gene repression? How about the expression of genes encoding enzymes (e.g. F3H, FLS, FGT) along the flavonol glycoside biosynthesis pathway?
(2) Subcellular localization of NCR-GFP should be better illustrated with more description and preferably high magnification of the microscopy images as well. Currently, it is difficult for general readers to distinguish between tonoplast and plasma membrane localization in Figure 7.

Minor issues
Line 47-48: CHS reaction include sequential condensation with malonyl-CoA prior to cyclization, not cyclization only.
Figure 1: Place the key structures in the pathway directly instead of separately.
Lines 164-165 and Fig S2: How about the expression of ARL1 and ARL3 in these transgenic lines?
Figure 3A: Label the HPLC traces on the graph directly.
Figure 4 legends: Should it be MdNCA1b?
Fig 4I: probably should be moved to supplementary data.
Fig. 5C: what was the control used?
Line 326-328: Phenylpropanoid pathway includes 4CL but not CHS
Line 347: Should be NCR proteins (not genes)
Figure 8: Label each panel with the corresponding enzyme name

Reviewer #3 (Comments for the Author):

In this manuscript, recombinant protein, kinetic and transient expression analyses have been used to confirm that MdNCR1 enzymes are capable of producing efficient production of phloretin both in vitro and in planta, which suggests a second route to phloridzin biosynthesis. This study contains some new findings and are valuable for the understanding of phloridzin biosynthesis in Malus. However, I think this study is lack of depth in experimental evidence, as well as the explanation of experimental design and analysis of results are insufficient. Therefore, major revision has to be done before it is considered for publication. Here are my major concerns:
1. The use of 'missing enzyme' in the title is quite confusing. I suspect that the authors intended to indicate that the enzyme has not been characterized yet, but 'missing' could easily be interpreted as 'the enzyme is lack in Malus leaves', which is inconsistent with the authors' findings. Please replace it with a more precise expression.
2. In the result section 'Characterization of recombinant MdNCR1b', whether it is possible to compare and summarize the catalytic ability of the enzyme to different types of substrates?
3. Line 232-233, "Further detailed analysis by LC-MS was performed using MdNCR1b because both proteins showed essentially identical activity." How did the authors come to this conclusion? Is it simply because of the results of transient overexpression? Is there more evidence? Moreover, why did MdNCR1b have been chosen but not MdNCR1a, since MdNCR1a instead of MdNCR1b was highlighted in the results section 'Modelling of apple NCR genes' and Figure S7? Please specify it.
4. Line 272-280, why did the authors have chosen MdNCR2a represent for MdNCR2, but MdNCR1a-c for MdNCR1 in the case that the function of MdNCR1c had not been comfirmed?

5. Why choose heterologous overexpression in N. benthamiana leaves over homologous overexpression? Homologous overexpression could be more convincing.
6. There are some mistakes should be corrected, the authors need to make careful modifications to the current manuscript. Such as, Line 139, '2′ 4 4′ 6′ tetrahydroxychalcone'; Line 686, it might be 'Figure 4. Activity of recombinant MdNCR1b in vitro' but not 'MdNCR1a in vitro'.
7. Figure 3(A): 'From top to bottom' means the peak or the baseline? Please describe clealy.
8. Figure S2(A), please add significance analysis.

RE: A naringenin chalcone carbon double-bond reductase is the missing enzyme in dihydrochalcone biosynthesis in apple leaves

Response to reviewer comments

Reviewer #1 (Comments for the Author):

In this research, the authors discovered the synthesis of Dihydrochalcones (DHCs) in apples via the phenylpropanoid pathway, highlighting MdHCDBR and MdNCR1a/b as pivotal components. While reducing MdHCDBR did not impact DHC levels, overexpressing MdNCR1a/b facilitated DHC production. Particularly, MdNCR1b efficiently transformed naringenin chalcone into phloretin. Suppressing MdNCR1 in apples decreased DHC levels, favoring the production of flavonol glycosides. Additionally, the likely vacuolar membrane localization of MdNCR1 sheds light on DHC regulation, offering promising implications for biotechnological advancements. I think the findings will be of interest to readers of Plant Physiology, especially those who are interested in phenylpropanoid pathway. I also think that the discoveries will provide valuable information into the field, however, at this time, the manuscript submitted seems to be premature and need to be improved.

1. My major comment is that the discussion section should be improved, it seems more a conclusion than a discussion section, and the amount of data and findings here described deserve further discussion.
Happy to add further paragraphs to the discussion.

2. Why should MdHCDBR, MdARL2, and their homologous genes all be knocked down through RNAi interference, and do they exhibit differential expression levels and potential functional redundancy among them? Please comment.
Figure S2A showed that *MdHCDBR* and *MdARL2* were downregulated in the transgenic ARL2n6 lines. Additional data on downregulation of *MdARL1* and *3* is now presented in Figure S2C.
The differential expression levels of the four genes are presented in Table S1.

The following paragraph discussing *MdHCDBR/*ARL and future opportunities has been added to the discussion.
The downregulation of MdHCDBR and MdARL2 by RNAi in transgenic apple leaves also downregulated two further related genes MdARL1 and MdARL3 (Figure S2). From this experiment we concluded that none of these four genes are responsible for the high concentrations of DHCs observed in apple leaves. Recombinant MdHCDBR expressed in E. coli can reduce p-coumaroyl CoA to dihydro-p-coumaroyl CoA (Ibdah et al., 2014), however no activity towards p-coumaroyl CoA was detected when the enzyme was expressed in yeast (Eichenberger et al., 2016; Caliandro et al., 2021). Three substrates for MdHCDBR expressed in yeast were identified, phenyl-3-buten-2-one, 4-hydroxybenzalacetone and p-coumaroyl aldehyde (Caliandro et al., 2021). Whether MdHCDBR and MdARL1-3 function to reduce these three substrates in planta is unclear. However, the availability of the transgenic Hn2 knockdown lines may allow target compounds and products to be identified in apple leaves and later in fruit.

3. How many NCR genes are present in apple, do they share similar functions, and are there any intriguing evolutionary events related to NCR genes in the Rosaceae family? Please elaborate further on this topic.
The following paragraph on the evolutionary events leading to neofunctionalization of NCR genes been added to the discussion.
The NCR1 clade specific for DHC production in Malus is associated with gene duplication and neofunctionalization of NCR genes that are present in Pyrus and other Rosaceae species. Other

genes involved in DHC synthesis (MdPGT1), and catabolism (polyphenol oxidase, PPO05 and PPO06; B-glucosidase, BGLU13), have also been reported to have undergone similar duplication and neofunctionalization (Elejalde-Palmett et al., 2018; Zhao et al., 2024). The enhanced capacity for DHC biosynthesis and catabolism is likely linked to improved apple resistance to pests and diseases such as two spotted mites, Valsa canker and powdery mildew (Zhou et al., 2019; Dare et al., 2020; Zhao et al., 2024). Although MdNCR2a did not produce DHCs when transiently overexpressed in N. benthamiana, the recombinant protein (if soluble and active) might allow identification of potential substrates for the broader NCR-related enzyme family. Transgenic downregulation of NCR-related genes in Pyrus and other Rosaceae would allow confirmation of their substrate preference as double bond reductases in planta. Although high concentrations of DHCs are typically associated with Malus leaves and fruit, other plants e.g. as Lithocarpus spp. (Qin and Liu, 2003; Sun et al., 2015) also accumulate DHCs in leaves. It will be of interest to see if the pathway to DHC production in other species occurs primarily via direct reduction of naringenin chalcone or via reduction of p-coumaroyl CoA.

Reviewer #2 (Comments for the Author):

The manuscript by Yauk et al. reported a key reaction step catalyzed by a novel enzyme naringenin chalcone reductase during the biosynthesis of dihydrochalcones. Using a combination of transient expression, in vitro enzyme assays, RNAi transgenic analyses, the authors established that phloretin is synthesized primarily through direct reduction of naringenin chalcone in apple leaves. Overall, the experiments were designed logically and data presented were quite conclusive. Nonetheless, there are a few issues that require clarification before the manuscript is acceptable.

Major issues:
(1) Downregulation of NCR genes in transgenic apple leaves resulted in decreased phlorizin production but increased levels of quercetin glycosides. Surprisingly, 4CL and CHS gene expressions were also down-regulated. As 4CL and CHS produce precursors for both flavonoids and dihydrochalcones, both types of metabolites should be reduced in abundance. In fact, the CHS downregulated line A9 showed 50% reduction in NCR1 expression with 90% reduction in both DHC and flavonoids (Line 295-297). The authors should address this issue in more detail.
The reduction in MdNCR1 expression appears to redirect flux away from the dominant DHC sidebranch in apple towards the flavonoid pathway which leads increased production of flavanol glycosides. However, as illustrated in Table 1, the redirection in flux is partial, with total DHC + other content reaching only 30-40% of wildtype leaves. Downregulation of CHS and 4CL may contribute to this overall reduction.
In the CHS downregulated line A9, the 90% reduction in DHCs and flavonoids results from the inability of the plant to produce the naringenin chalcone precursor that is required for production of DHCs by NCRs and for production of naringenin by CHI. The reduction in NCR expression in the CHS downregulated line A9 may be a response to reduction in available naringenin chalcone precursor or to the increase in compounds lower in flavonoid pathway.
We have added more details on these points to the results section.

For example, was the RNAi downregulation in their transgenic apple specific to NCR genes only?
RNAi downregulation of NCR genes (as seen in Figure 6A) appeared specific to the four MdNCR genes targeted (MdNCR1a-c and NCR101). The RNAi did not downregulate expression of MdNCR2, HCDBR or MdARL1-3.

Was the reduced phlorizin level directly caused by NCR gene repression?

The reduced phlorizin level appeared directly related to levels of NCR1 gene expression. In the four lines showing >90% reduction in NCR1 gene expression (Figure 6A), phlorizin levels were also reduced >90% (Figure 6B). Lines showing intermediate levels of NCR1 gene repression (Figure S5A) showed intermediate reductions in phlorizin levels (Figure S5B). Reductions in MdCDBR and MdARL1-3 expression (Figure S2A, B) did not affect phlorizin accumulation (Figure S2C).

How about the expression of genes encoding enzymes (e.g. F3H, FLS, FGT) along the flavonol glycoside biosynthesis pathway?
Additional data on expression of F3H and FGT (and ARL1 and 3) has been added to Figure 6D/Figure S5C and described in the results. FLS expression was not detected.

(2) Subcellular localization of NCR-GFP should be better illustrated with more description and preferably high magnification of the microscopy images as well. Currently, it is difficult for general readers to distinguish between tonoplast and plasma membrane localization in Figure 7.
Higher resolution figures have been provided allowing for enlarged views and more description of the figure has been added in the results section.

Minor issues
Line 47-48: CHS reaction include sequential condensation with malonyl-CoA prior to cyclization, not cyclization only.
This sentence has been simplified and shortened.
Figure 1: Place the key structures in the pathway directly instead of separately.
Structures have been added to the pathway.
Lines 164-165 and Fig S2: How about the expression of ARL1 and ARL3 in these transgenic lines?
Additional data on downregulation of ARL1 and 3 is now presented in Figure S2C and described in the results.
Figure 3A: Label the HPLC traces on the graph directly.
The traces have been labelled in the figure and the colours described in the legend for clarity.
Figure 4 legends: Should it be MdNCA1b?
This has been corrected.
Fig 4I: probably should be moved to supplementary data.
Happy to move the figure if required to make space for other data by the editor.
Fig. 5C: what was the control used?
The control was wildtype (WT) 'Royal Gala' controls. This has been added to the legend.
Line 326-328: Phenylpropanoid pathway includes 4CL but not CHS.
This has been changed to flavonoid pathway here and throughout the manuscript as appropriate.
Line 347: Should be NCR proteins (not genes)
This has been corrected.
Figure 8: Label each panel with the corresponding enzyme name
Labels have been added to each picture.

Reviewer #3 (Comments for the Author):

In this manuscript, recombinant protein, kinetic and transient expression analyses have been used to confirm that MdNCR1 enzymes are capable of producing efficient production of phloretin both in vitro and in planta, which suggests a second route to phloridzin biosynthesis. This study contains some new findings and are valuable for the understanding of phloridzin biosynthesis in Malus. However, I think this study is lack of depth in experimental evidence, as well as the explanation of experimental design and analysis of results are insufficient. Therefore, major revision has to be done

before it is considered for publication. Here are my major concerns:

1. The use of 'missing enzyme' in the title is quite confusing. I suspect that the authors intended to indicate that the enzyme has not been characterized yet, but 'missing' could easily be interpreted as 'the enzyme is lack in Malus leaves', which is inconsistent with the authors' findings. Please replace it with a more precise expression.
The title has been changed to 'Naringenin chalcone carbon double-bond reductases are required for dihydrochalcone biosynthesis in apple leaves'.
In the abstract the wording has been clarified by adding the word required to the last sentence. 'Identifying the missing enzyme required for DHC production provides opportunities for the manipulation of DHC content in apple and other fruit as well in other applications, e.g. biofermentation and biopharming'.

2. In the result section 'Characterization of recombinant MdNCR1b', whether it is possible to compare and summarize the catalytic ability of the enzyme to different types of substrates?
The focus was on analysis of substrates that are found in apple leaves. The three additional substrates that MdNCR1b shows activity towards in Figure 4I (isoliquiritegenin, p-hydroxybenzalacetone, 1-penten-3-one) are not found in apple leaves so were not investigated in further detail.

3. Line 232-233, "Further detailed analysis by LC-MS was performed using MdNCR1b because both proteins showed essentially identical activity." How did the authors come to this conclusion? Is it simply because of the results of transient overexpression? Is there more evidence? Moreover, why did MdNCR1b have been chosen but not MdNCR1a, since MdNCR1a instead of MdNCR1b was highlighted in the results section 'Modelling of apple NCR genes' and Figure S7? Please specify it.

MdNCR1a–c show >98% amino acid identity and exhibit essentially identical activity when transiently overexpressed *in planta*. Therefore, MdNCR1b was selected for detailed biochemical analysis. This rationale has been stated more clearly in the results.
Modelling was performed on MdNCR1a-c. The text has been corrected to state this clearly as well.

4. Line 272-280, why did the authors have chosen MdNCR2a represent for MdNCR2, but MdNCR1a-c for MdNCR1 in the case that the function of MdNCR1c had not been confirmed?
Data on the activity of *MdNCR1c* has been added to Figure 3B + Figure S3C and described in the results.

5. Why choose heterologous overexpression in N. benthamiana leaves over homologous overexpression? Homologous overexpression could be more convincing.
Heterologous over-expression in N. benthamiana is a rapid and reliable screen for enzyme activity. Given the high levels of phlorizin existing in apple leaves, overexpression of the NCR gene would be unlikely to increase phlorizin levels further.

6. There are some mistakes should be corrected, the authors need to make careful modifications to the current manuscript. Such as, Line 139, '2’ 4 4’ 6’ tetrahydroxychalcone';
Corrected to 2’, 4, 4’, 6’-tetrahydroxychalcone.

Line 686, it might be 'Figure 4. Activity of recombinant MdNCR1b in vitro' but not 'MdNCR1a in vitro'.
Corrected to MdNCR1b.

7. Figure 3(A): 'From top to bottom' means the peak or the baseline? Please describe clearly.
The traces have been labelled in the figure and the colours described in the legend for clarity.

8. Figure S2(A), please add significance analysis.
Significance analysis has been added to Figure S2A.

12-Aug-2024

Dr. Ross G. Atkinson
New Zealand Institute for Plant and Food Research Ltd
Fruit Quality
Auckland, n/a 1141
New Zealand

RE: Naringenin chalcone carbon double-bond reductases are required for dihydrochalcone biosynthesis in apple leaves

Dear Dr. Ross Atkinson:

We are pleased to accept your manuscript for publication in the Plant Physiology. This acceptance is contingent on revision based on the comments of our reviewer #3.

Please highlight all changes and include a detailed annotation to changes to the text, with line numbers, and noting your responses to the comments.

To submit your revised manuscript, click:

Link Not Available

If you cannot return the revised manuscript within 8 weeks of receipt, please let us know. Otherwise, we will assume that you have elected not to revise the manuscript and are withdrawing it.

Thank you for allowing us to review your work. We look forward to hearing from you soon.

Sincerely,

Editor, Plant Physiology

-- Reviewer comments:
Reviewer #1 (Comments for the Author):

All comments were addressed

Reviewer #2 (Comments for the Author):

The authors have addressed all my concerns satisfactorily.

Reviewer #3 (Comments for the Author):

In the revised manuscript, the author has supplemented many details, thereby enhancing the coherence of the text in terms of sentence structure and content logic. Additionally, some of the previous concerns have been elucidated in a more compelling manner. However, there are two other aspects that warrant further refinement by the author:
1. In the response, the author mentioned, "Given the high levels of phlorizin existing in apple leaves, overexpression of the NCR gene would be unlikely to increase phlorizin levels further. " Has this hypothesis been tested to confirm that overexpression in apple leaves is not obvious? As the author stated in the Introduction, phloretin is a distinctive active compound found in apples. If the content of leaves is too high to be suitable for overexpression materials, other tissues of apples, such as fruits, may also be considered.
2. In the results of subcellular localization of NCRs, an empty control group was lacking.

Re: PP2024-RA-00532R1. Naringenin chalcone carbon double-bond reductases are required for dihydrochalcone biosynthesis in apple leaves by Yauk *et al.*

Response to **Reviewer #3** comments:

1. In the response, the author mentioned, Given the high levels of phlorizin existing in apple leaves, overexpression of the NCR gene would be unlikely to increase phlorizin levels further. " Has this hypothesis been tested to confirm that overexpression in apple leaves is not obvious? As the author stated in the Introduction, phloretin is a distinctive active compound found in apples. If the content of leaves is too high to be suitable for overexpression materials, other tissues of apples, such as fruits, may also be considered.

Overexpression of the NCR construct in apple is a good idea and we have carefully considered options to achieve this.

In New Zealand, due to compliance regulations, we are unable to perform experiments where genes are overexpressed from viral expression vectors.

In our hands, transient overexpression of constructs in apple leaves and fruit using Agrobacterium infiltration is inefficient and unreliable. NCR constructs overexpressed in leaves would also likely be cosuppressed, given the high level of NCR expression in leaves.

The definitive answer is to make stable transgenic plants. As part of exemplification of PFR's patent on the NCR gene (Atkinson & Yauk, 2024), we are making stable transgenic apple plants that will attempt to overexpress the NCR gene in fruit to increase DHC levels. However, increasing DHC levels is not only dependent on overexpression of the NCR construct, but to substrate availability as well. It will be several years before any fruiting transgenic apple plants will be available for study.

Atkinson RG, Yauk YK (2024) REDUCTASES, POLYNUCLEOTIDES ENCODING THESE AND METHODS OF USE. Australian provisional patent application 2024900937.

2. In the results of subcellular localization of NCRs, an empty control group was lacking.
An 'empty' uninfiltrated control was performed as a negative control along with 35S-GFP infiltrations as positive controls. The results from these experiments established the localization via confocal microscopy was working as expected.

We have included images of the 35S-GFP infiltrations in a new supplemental figure (**Figure S6**).

We have also reworded the results to clarify our findings (Line 372-373).
No GFP or RFP fluorescence was detected in uninfiltrated tissues. For the positive control 35S-GFP construct, signals were found throughout the cell (**Figure S6**).

04-Sep-2024
Dr. Ross G. Atkinson
New Zealand Institute for Plant and Food Research Ltd
Fruit Quality
Auckland, n/a 1141
New Zealand

MSID: PP2024-RA-00532R2
MS TITLE: Naringenin chalcone carbon double-bond reductases are required for dihydrochalcone biosynthesis in apple leaves

Dear Dr. Ross Atkinson:

I am pleased to inform you that your revised manuscript "Naringenin chalcone carbon double-bond reductases are required for dihydrochalcone biosynthesis in apple leaves" has been accepted for publication in Plant Physiology. Your manuscript will be checked for consistency with journal formatting and image standards by one of our Science Editors, who will contact you shortly. They may have a number of queries and suggestions for you. Please keep in mind that they work to increase the online visibility of your article and its attraction for readers from the widest of backgrounds. You will have the opportunity to review any revisions, and I hope you will work constructively with them to get the most out of your research. You will also have the opportunity to review all changes when you receive the author proofs for your article.

Once validated for publication, your article will be sent to a Science Editor for a final review of your manuscript and source files. After your final files are uploaded, your article will appear in the next available issue and the online Preview version of your article will be posted after your signed Copyright and Checklist forms and final source files are received.

Please be sure to include the following in your manuscript file:
Harvard style citations
List of all author contributions and funding information at the beginning of manuscript after the titles and author list.
List all supplemental materials associated with the article, with titles, at the end of the ms, before the references and acknowledgments section.
Complete Figure Legends for all figures appearing in the article listed before the Literature Cited.

LICENSE INFORMATION Shortly after your paper is sent to production, you will receive an email from our publisher's author support team, **SciPris**, with information on article processing charges, as well as the types of Open Access license you would be able to purchase. The accepted version of your manuscript will not appear on our Advance Articles page until the license is selected and signed, so please look out for an email from SciPris. For more information see the Article Publication Fees section of our author guidelines: https://academic.oup.com/plcell/pages/General-Instructions

Finally, we encourage your submission of artwork for the journal cover. If there is an image or illustration related to your paper that you would like to have considered, please email it in .pdf or .tif format to clowe@aspb.org. An image title, brief, 2-3 sentence description of the image, and the name of the person credited for the image are also encouraged. If your image is selected to appear on the online cover of the journal, you will be contacted with the scheduled issue date and further information.

Thank you and congratulations on your Plant Physiology paper!

Sincerely,
Editor, Plant Physiology

=====
FIRST AUTHOR PROFILES
=====

We recognize the hard work that goes into being First Author of a Plant Physiology paper by publishing a "First Author Feature" for each First Author alongside your paper.

If the first author(s) would like to submit information for a feature on Facebook and Twitter, fill out the form by clicking on the link below:

Plant Physiology First Author Feature Form: https://forms.office.com/r/ZBCGZfhm8F
If you cannot access the form through the link above, please contact Rachel at rbelsky@aspb.org.

To upload an image of yourself to accompany your profile, please put your photo in the correct journal folder at the link below. You must label your image: Author First Name Last Name Article Title.

First Author Profile Image Dropbox: https://www.dropbox.com/scl/fo/xz029p7qjr2jml2rp2kmq/h?dl=0&rlkey=6fkxcaqmxx35ap152b2osy1fz

=====
IMPORTANT REMINDER: PEER REVIEW REPORTS
=====

If you opted to publish a peer review report along with your article during the original submission process, it will be prepared by the editorial staff and publicly posted with your manuscript, inside the zip file that contains any other supplemental material. As a reminder, the peer review report is a public record of all comments from editors and reviewers, as well as your prior responses, as you received them in the decision letters for each draft of your manuscript. If you agreed to publish this report and have changed your mind, or are not sure if you selected this option, please contact the editorial office as soon as possible before signing the license agreement from our publisher.

-- Reviewer comments:
